# Cost and Effectiveness of Blended Versus Standard Cognitive Behavioral Therapy for Outpatients With Depression in Routine Specialized Mental Health Care: Pilot Randomized Controlled Trial

**DOI:** 10.2196/14261

**Published:** 2019-10-29

**Authors:** Lisa Catharine Kooistra, Jenneke Elize Wiersma, Jeroen Ruwaard, Koen Neijenhuijs, Joran Lokkerbol, Patricia van Oppen, Filip Smit, Heleen Riper

**Affiliations:** 1 Department of Clinical, Neuro and Developmental Psychology, Clinical Psychology Section Vrije Universiteit Amsterdam Amsterdam Netherlands; 2 Department of Research and Innovation GGZ in Geest/Amsterdam University Medical Center, VU University Medical Center Amsterdam Netherlands; 3 Amsterdam Public Health Research Institute Amsterdam University Medical Center, VU University Medical Center Amsterdam Netherlands; 4 Cancer Center Amsterdam Amsterdam University Medical Center, VU University Medical Center Amsterdam Netherlands; 5 Center of Economic Evaluation Trimbos Institute (Netherlands Institute of Mental Health and Addiction) Utrecht Netherlands; 6 Department of Psychiatry GGZ in Geest/Amsterdam University Medical Center, VU University Medical Center Amsterdam Netherlands; 7 Department of Epidemiology and Biostatistics Amsterdam University Medical Center, VU University Medical Center Amsterdam Netherlands

**Keywords:** depression, blended cognitive behavioral therapy, specialized mental health care, cost-effectiveness, randomized controlled trial

## Abstract

**Background:**

Cognitive behavioral therapy (CBT) is an effective treatment, but access is often restricted due to costs and limited availability of trained therapists. Blending online and face-to-face CBT for depression might improve cost-effectiveness and treatment availability.

**Objective:**

This pilot study aimed to examine the costs and effectiveness of blended CBT compared with standard CBT for depressed patients in specialized mental health care to guide further research and development of blended CBT.

**Methods:**

Patients were randomly allocated to blended CBT (n=53) or standard CBT (n=49). Blended CBT consisted of 10 weekly face-to-face sessions and 9 Web-based sessions. Standard CBT consisted of 15 to 20 weekly face-to-face sessions. At baseline and 10, 20, and 30 weeks after start of treatment, self-assessed depression severity, quality-adjusted life-years (QALYs), and costs were measured. Clinicians, blinded to treatment allocation, assessed psychopathology at all time points. Data were analyzed using linear mixed models. Uncertainty intervals around cost and effect estimates were estimated with 5000 Monte Carlo simulations.

**Results:**

Blended CBT treatment duration was mean 19.0 (SD 12.6) weeks versus mean 33.2 (SD 23.0) weeks in standard CBT (*P*<.001). No significant differences were found between groups for depressive episodes (risk difference [RD] 0.06, 95% CI −0.05 to 0.19), response to treatment (RD 0.03, 95% CI −0.10 to 0.15), and QALYs (mean difference 0.01, 95% CI −0.03 to 0.04). Mean societal costs for blended CBT were €1183 higher than standard CBT. This difference was not significant (95% CI −399 to 2765). Blended CBT had a probability of being cost-effective compared with standard CBT of 0.02 per extra QALY and 0.37 for an additional treatment response, at a ceiling ratio of €25,000. For health care providers, mean costs for blended CBT were €176 lower than standard CBT. This difference was not significant (95% CI −659 to 343). At €0 per additional unit of effect, the probability of blended CBT being cost-effective compared with standard CBT was 0.75. The probability increased to 0.88 at a ceiling ratio of €5000 for an added treatment response, and to 0.85 at €10,000 per QALY gained. For avoiding new depressive episodes, blended CBT was deemed not cost-effective compared with standard CBT because the increase in costs was associated with negative effects.

**Conclusions:**

This pilot study shows that blended CBT might be a promising way to engage depressed patients in specialized mental health care. Compared with standard CBT, blended CBT was not considered cost-effective from a societal perspective but had an acceptable probability of being cost-effective from the health care provider perspective. Results should be carefully interpreted due to the small sample size. Further research in larger replication studies focused on optimizing the clinical effects of blended CBT and its budget impact is warranted.

**Trial Registration:**

Netherlands Trial Register NTR4650; https://www.trialregister.nl/trial/4408

**International Registered Report Identifier (IRRID):**

RR2-10.1186/s12888-014-0290-z

## Introduction

Several evidence-based pharmacological and psychological treatments have been developed for major depressive disorder (MDD) [1,2]. Within the domain of psychotherapy, there is an especially large body of evidence supporting the efficacy of cognitive behavioral therapy (CBT) [3]. Unfortunately, there is a discrepancy between treatment availability and treatment demand [4]. This can partly be explained by increasingly insufficient mental health care budgets, which limit the availability of trained psychotherapists who can provide evidence-based treatments [5]. As a consequence, patients with severe symptoms often do not receive treatment, or they have to be placed on waiting lists rather than having immediate access to specialized depression care [4,6]. Therefore, there is a high need for efficient and cost-effective mental health care to manage this problem [7].

Web-based (online) psychotherapy is often cited as a promising way to reduce treatment costs and increase treatment availability for common mental disorders [8-11]. For example, it may lower the required therapist time per patient [12]. The efficacy of several online treatments has been demonstrated for the treatment of depression when compared with control groups [13-15]. Evidence suggests that therapist-guided online treatment can be equally as effective as standard face-to-face therapy [16,17]. The cost-effectiveness of guided online treatment has been less well studied. An individual patient data meta-analysis, combining data from five randomized controlled trials (RCTs), suggested that guided online treatment is not yet cost-effective compared with control conditions [18].

To date, most studies have focused on community samples and self-referred participants. Less is known about the costs and effects of online therapy in routine specialized mental health care for patients with more severe or complex depression profiles. This patient group is likely to require more personalized treatment, such as monitoring of suicidal ideation or addressing comorbid disorders [16]. The integration of online and face-to-face treatment into a blended treatment format could be a promising way to address these matters [19-23]. Blended treatment allows therapists to closely monitor their patients, both in face-to-face sessions at the clinic and in an online environment. At the same time, it is thought to retain the positive aspects associated with online treatment, such as lower costs compared with standard treatment, reduction of therapist time, and increased patient self-management [19,24,25].

Although several studies are in progress, current evidence for blended treatment is limited [26-29]. Most studies to date have evaluated the online component as an add-on to standard care rather than offering an integrated blended treatment protocol [30-35]. Results of these studies are promising and indicate that blended treatment may be a viable treatment option. However, the treatment format limits the margin for cost-effectiveness because therapist time is not reduced and overall treatment dosage may even increase [18,36]. Recently, Thase and colleagues [25] used a more integrated approach to blended treatment. Therapist time was limited by combining nine online sessions with twelve 25-minute (instead of 50-minute) face-to-face sessions. Compared with CBT (n=77), blended CBT (n=77) led to noninferior results on the Hamilton Depression Rating Scale (HAM-D) in medication-free adults with depression, when provided in two university clinics.

This study compares the costs and effects of integrated blended CBT with standard CBT when provided to depressed patients in the acute phase of treatment in specialized mental health care. The blended treatment aimed to replace half of face-to-face treatments with online sessions, and thereby shorten treatment duration when compared with standard CBT for MDD [22]. The study examines whether blended CBT has the potential to lead to comparable clinical effects as standard CBT at lower costs. The study was designed as a pilot study with the aim to guide further development of blended CBT and inform future research on feasibility, effectiveness, and cost-effectiveness of blended CBT for depression in outpatient specialized mental health care.

## Methods

### Study Design and Participants

This pilot study was designed as an RCT in three mental health care organizations in the Netherlands, at five outpatient treatment locations. Blended CBT was compared with standard CBT for depression. Outcomes were measured before treatment allocation at baseline and 10, 20, and 30 weeks after start of treatment. Adult patients with a *Diagnostic and Statistical Manual of Mental Disorders* (Fourth Edition, Text Revision; *DSM-IV-TR*) diagnosis [37] of MDD, who were indicated for individual CBT by the local intake staff, were recruited during the intake procedure. In the Netherlands, patients can be referred to specialized services when they do not respond to treatment in primary mental health care [38]. Therefore, patients in this trial were likely to have complex or severe clinical profiles and to have undergone some form of pharmacotherapy or short-term psychotherapy before this study.

Exclusion criteria were inadequate proficiency in the Dutch language; no valid email address or no computer with internet access; current psychotic disorder, bipolar disorder, or substance dependence; or high risk for suicide (current plans). Patients with current substance dependence could participate in the trial if they reported abstinence and had been treated at a center specialized in treatment of substance abuse for at least a month before random allocation.

Diagnoses and suicidal ideation were assessed with the Mini-International Neuropsychiatric Interview Plus (MINI-Plus) [39,40]. Assessors were trained research assistants with master’s degrees in psychology. The study protocol has been published [41]. The trial was registered in the Netherlands Trial Register (registration number NTR4650) and approved by the Medical Ethics Committee of the VU University Medical Center Amsterdam (registration number 2014.191).

### Randomization and Masking

Patients were randomly allocated to blended CBT or standard CBT when they met the inclusion criteria, provided a signed informed consent form, and completed the full baseline assessment. Allocation was stratified by mental health care center using a computer-generated random number table. An independent researcher conducted the randomization. Patients and therapists were aware of group allocation, but allocation was concealed from outcome assessors.

### Interventions

Both interventions consisted of cognitive behavioral depression treatment. The content of CBT was based on the standard treatment manual [42-44], which recommends 15 to 20 weekly sessions focusing on psychoeducation, behavioral activation, cognitive restructuring, and relapse prevention. As part of the treatment, patients in both treatment groups were encouraged to fill in the 16-item Quick Inventory of Depressive Symptomatology Self-Report on a weekly basis to allow patients and their therapists to monitor change in depression severity [45]. Patients who completed 14 sessions or more (75% of 18 sessions) were considered treatment completers. Parallel pharmacological treatment was allowed.

Blended CBT consisted of 10 face-to-face sessions at the specialized mental health care center and 9 Web-based (online) sessions that patients worked through at home. Therapists and patients had personal, password-protected accounts on the Web-based treatment platform. Therapists were trained in the use of the Web-based treatment platform and received a treatment manual. Patients received information on how to work with the platform in the first face-to-face session. After each online session, therapists provided online therapeutic feedback regarding content and progress. Therapists could let patients repeat an online session if this was warranted. The blended CBT protocol aimed to provide one face-to-face session, one online session, and one online feedback message per week over 10 weeks, starting with a face-to-face session. The intervention was semistructured, offering online sessions in a fixed order and providing therapists with a protocol for each face-to-face session. Therapists were allowed to personalize face-to-face sessions to each patient’s individual needs and situation in terms of themes and techniques. More detailed information on the format and content of blended CBT can be found elsewhere [22,41].

The standard CBT group received individual face-to-face cognitive behavioral depression treatment at the clinic in accordance with routine care procedures. The content of standard CBT was comparable to the content of blended CBT. Therapists were advised to plan weekly sessions but were allowed to deviate from the treatment manual when necessary. Based on the guideline, standard CBT was expected to consist of 18 sessions on average, provided over approximately 20 weeks. However, duration of treatment could vary per patient.

### Outcomes

The primary clinical outcome was self-reported depression severity at 10, 20, and 30 weeks after the start of treatment, as measured by a Web-based version of the Inventory of Depressive Symptomatology Self-Report (IDS-SR_30_) [46-49]. Within the cost-effectiveness framework, the IDS-SR_30_ scores were used to assess treatment response based on the reliable change index (RCI) [50]. Baseline IDS-SR_30_ scores were subtracted from follow-up scores and then divided by the standard error of the difference scores (SE 4.78, Cronbach alpha=.84). Treatment response (reliable change) was coded as absent (RCI≥−1.96) versus present (RCI<−1.96). Remission was defined as the combination of an RCI less than −1.96 and an IDS-SR_30_ score less than 13, indicating no depression severity on the IDS-SR_30_ severity index [46,51].

Clinician-rated outcomes included the presence of a depressive episode at 10, 20, and 30 weeks after the start of treatment and during follow-up. Depression diagnosis was assessed at 10, 20, and 30 weeks after the start of treatment during a telephone interview with section A of the MINI-Plus diagnostic interview [39,40] by trained research assistants who were blinded to treatment allocation. At baseline and 30-week follow-up, all sections of the MINI-Plus interview were administered during a face-to-face or telephone interview to assess comorbid diagnoses.

To calculate quality-adjusted life-years (QALYs), the EQ-5D three-level version [52] questionnaire was administered online at all four time points. QALYs gained over the 30-week study period were estimated by linearly interpolating EQ-5D utility over the time points and correcting for time. Utility scores were based on the Dutch tariff [53].

Self-reported resource use in the 4 weeks before each assessment was measured with a Web-based version of the Trimbos/iMTA questionnaire for Costs associated with Psychiatric Illness (TiC-P) [54]. The TiC-P includes questions on (1) the number of visits to various health care providers in primary and specialized care settings; (2) medication use for sleep, depression, and anxiety; (3) help from friends and family; and (4) absenteeism and reduced work productivity (presenteeism) in paid and unpaid work. For the main economic evaluation, costs were estimated from a societal perspective*,* which included all costs measured by the TiC-P. The health care provider perspective only included direct medical costs. A full overview of all cost categories can be found in Multimedia Appendix 1. Costs were computed by multiplying the number of units (contacts, visits, sessions) by the standard unit cost prices (for the year 2014) as reported in the most recent Dutch guideline for economic evaluations [55]. When costs were not included in the latest version of the manual, the cost price was derived from the previous guideline [56] and indexed to the year 2014. Medication prices were retrieved from the National Healthcare Institute (Zorginstituut Nederland) (Z-index [57]). For each prescription, the standard dispensing fee for pharmacists of €6 was added. For blended CBT patients, costs associated with the actual number of online feedback messages received were added, based on an estimated 30 minutes of therapist time per feedback message. Cumulative costs over the 30-week study period were estimated using linear interpolation.

### Statistical Analyses

For descriptive purposes, clinical and cost outcomes were first examined in separate linear mixed-effect models. To account for missing data and the correlation between follow-up time points, linear mixed-effect models with restricted maximum likelihood were used to estimate the treatment effects, EQ-5D utility gained, and cumulative costs across time. The models included both fixed and random effects, which allowed for estimation on a patient level of how far each patient diverged from the fixed (group level) effect over time [58,59]. Main outcome measures (ie, IDS-SR depression severity, reliable change, MINI depression status, EQ-5D QALYs, and costs) were evaluated in separate mixed models. Time was included as a categorical variable (0, 10, 20, and 30 weeks). A logistic mixed-effects model was fitted for diagnosis of depressive episodes (present versus absent) and reliable improvement of depression severity (improvement versus no improvement). In these models, the random intercept was dropped from the model because all patients started at the same baseline value (a current diagnosis of depression).

For the cost-effectiveness analyses, baseline adjusted linear mixed-effect models were estimated with group as the independent variable and societal costs, direct medical costs, and reliable change in depression severity as the dependent variables and a random effect across individuals. For dichotomous outcomes, linear mixed-effect models were fitted to estimate risk difference and calculate the incremental cost-effectiveness ratio (ICER). EQ-5D QALYs gained during the study were used as the dependent variable for the cost-utility analysis. Within the cost-effectiveness and cost-utility framework, time was not included in the regression models, rendering one estimate for cost, effectiveness, or utility for all follow-ups. Societal costs during the study period were used as the main cost outcome. Direct health care costs were examined as a separate cost outcome to conduct the health-economic evaluation from the health care system perspective. Uncertainty intervals of 95% around linear mixed-effect model regression estimates for group were estimated with 5000 probabilistic Monte Carlo simulations, running all mixed models simultaneously.

The resulting pairs of cost and effect or utility estimates were plotted on cost-effectiveness and cost-utility planes. The y-axis represented relative costs, and the x-axis represented relative effects associated with blended CBT versus standard CBT. The axes divide the plane into four quadrants. In the northeast quadrant, blended CBT was more expensive and more effective than standard CBT. In the northwest quadrant, blended CBT was more expensive and less effective than status quo (standard CBT), meaning that blended CBT was dominated by standard CBT. In the southwest quadrant, blended CBT was less expensive and less effective than standard CBT. In the southeast quadrant, blended CBT was less expensive and more effective than standard CBT, which indicated that blended CBT dominated standard CBT. Cost-effectiveness acceptability curves were estimated to assess the probability of blended CBT being cost-effective given various willingness-to-pay ceilings.

The mixed models were estimated using the lmer and glmer functions from the lme4 package (version 1.1-15) [60] using R software (version 3.4.4) [61]. Combined cost-effectiveness and cost-utility analyses were performed in Stata version 14.2 (StataCorp LP, College Station, TX, USA).

Two sensitivity analyses were conducted. First, a linear mixed-effect model was estimated for societal costs excluding the costs of in-patient care (both in general hospitals and mental health care). Although these events are rare, they can act as influential outliers due to the high costs associated with in-patient care. Second, direct nonmedical costs associated with absenteeism and presenteeism were evaluated as a separate cost outcome to assess their contribution to the overall societal costs as evaluated in the main analysis.

### Power

Sample size estimation was based on the probability of blended CBT being cost-effective in comparison with standard CBT for various willingness-to-pay ceilings (ie, the maximum additional financial contribution society is willing to invest to gain one more unit of treatment effect) [62]. Through a simulation study, the impact of different sample sizes on the stability of the cost-effectiveness acceptability curve was determined [62]. Using realistic fixed estimates of the mean and standard deviation of effects and the costs on the population level (based on Hakkaart-van Roijen et al [63]), a large number of trials were simulated in which sample sizes were systematically varied between n=10 to n=500 per group. At a sample size of n=75 per group, the probability estimates converged to acceptable 75% values within the relevant range of willingness-to-pay ceilings. Therefore, this study aimed for a sample size of N=150.

## Results

### Overview

**Figure 1:**
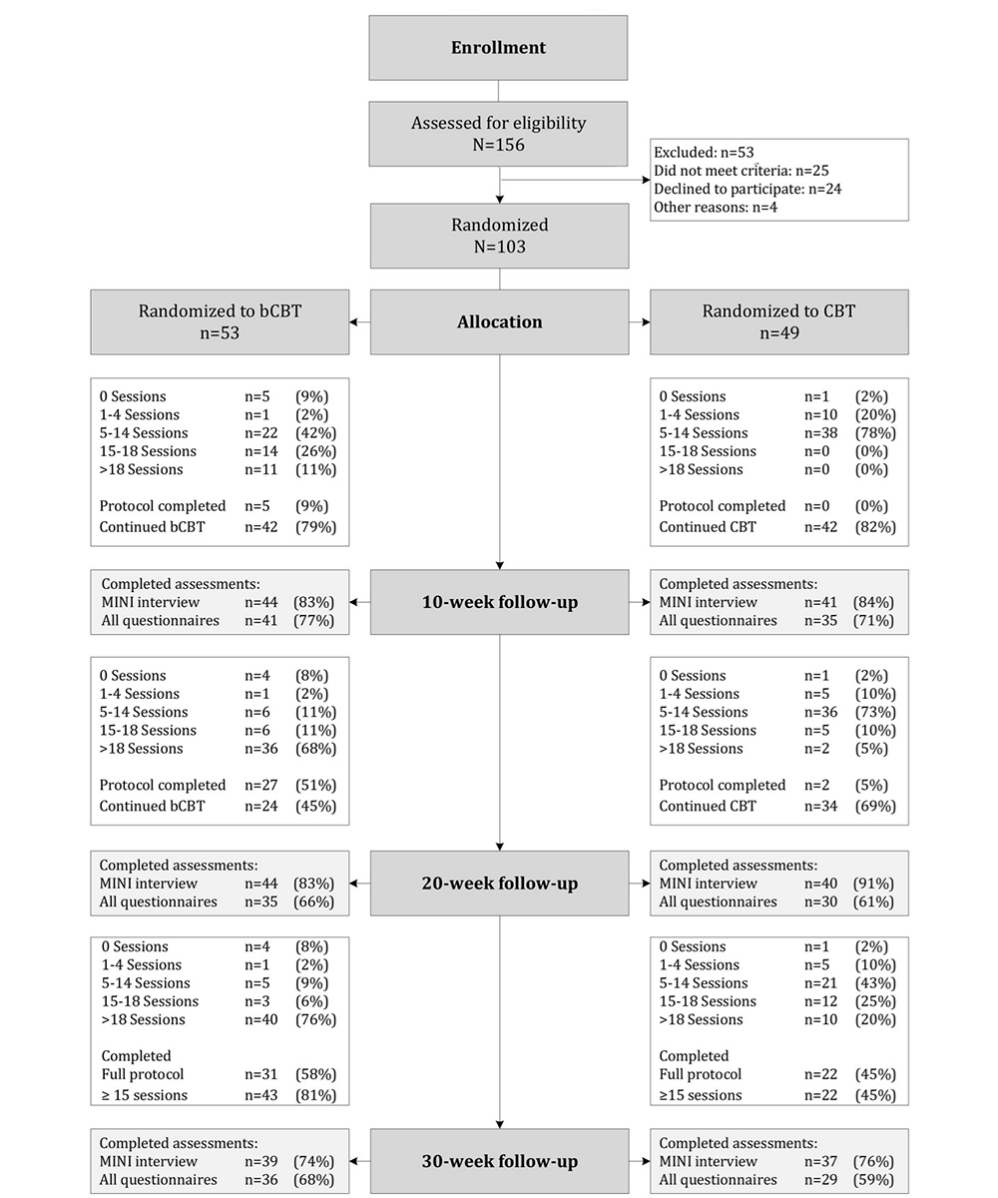
Flowchart. bCBT: blended cognitive behavioral therapy; CBT: standard cognitive behavioral therapy.

Between August 2014 and May 2016, 103 patients were randomized to blended CBT (n=54) or standard CBT (n=49). One patient withdrew from the study before commencing treatment due to starting another treatment elsewhere. The trial profile is presented in Figure 1. The full intention-to-treat sample consisted of 102 patients (blended CBT: n=53; standard CBT: n=49). This sample size was smaller than the initial goal of 150 patients [41]. The recruitment and screening of sufficient patients to randomize 150 patients proved to be unfeasible within the fixed time frame of this study. Fewer patients were referred to specialized services than expected. This was partly due to a nationwide reorganization of Dutch mental health care, which meant that a larger proportion of depressed patients were treated in primary mental health care rather than specialized care.

Information on patients’ baseline demographic characteristics, clinical profiles, and costs are presented in Table 1. A notable number of patients (69 of 102, 68%) were diagnosed with at least one other *DSM-IV* disorder in addition to MDD, 60% (61 of 102) reported suicidal ideation, and 28% (29 of 102) had attempted suicide at some point in their lives. Patients in blended CBT showed a higher baseline depression severity (45.2 versus 41.5), lower mean utility score (0.36 versus 0.46), and higher direct medical costs (€989 versus €439) in the 4 weeks before baseline than patients in standard CBT.

**Table 1:** Sample characteristics of participants at baseline (N=102).

Patient characteristics			Blended CBT^a^ (n=53)	Standard CBT (n=49)	Total (N=102)
**Demographic**					
	Gender (female), n (%)		35 (66)	29 (60)	64 (63)
	Age (years), mean (SD)		39.3 (11.3)	38.1 (10.6)	38.8 (10.9)
	In a relationship, n (%)		30 (57)	30 (61)	60 (59)
	**Education, n (%)**				
		Low	6 (11)	2 (4)	8 (8)
		Middle	30 (57)	33 (67)	63 (62)
		High	17 (32)	14 (29)	31 (30)
	Employed, n (%)		31 (59)	28 (57)	59 (58)
	Nationality (Dutch), n (%)		48 (91)	47 (96)	95 (93)
**Clinical characteristics**					
	Blended CBT treatment preference, n (%)		33 (62)	33 (67)	66 (65)
	Any comorbidity,^b^ n (%)		38 (72)	31 (63)	69 (68)
	Anxiety disorders, n (%)		30 (57)	25 (51)	55 (54)
	Other disorders,^c^ n (%)		25 (47)	23 (47)	48 (47)
	Comorbid disorders,^d^ mean (SD)		1.8 (1.7)	1.8 (1.9)	1.8 (1.7)
	MDD^e^ duration in months, mean (SD)		73.7 (73.2)	80.2 (69.8)	76.9 (70.9)
	Depression (IDS-SR)^f^, mean (SD)		45.2 (12.1)	41.5 (11.6)	43.4 (11.9)
	**Severity (IDS-SR), n (%)**				
		Mild/moderate	17 (32)	22 (45)	39 (38)
		Severe/very severe	36 (68)	27 (55)	64 (63)
	Utility scores, mean (SD)		0.36 (0.29)	0.46 (0.31)	0.41 (0.30)
**Prior treatment (4 weeks), n (%)**					
	Antidepressant medication		36 (68)	29 (59)	65 (64)
	Psychological treatment		44 (83)	42 (86)	86 (84)
**Costs (€, TiC-P^g^, 4 weeks), mean (SD)**					
	Direct medical		989 (1626)	439 (398)	725 (1230)
	Direct nonmedical		428 (802)	246 (526)	339 (685)
	Indirect nonmedical		796 (1629)	716 (1610)	757 (1613)
	Societal costs		2205 (2615)	1401 (1762)	1819 (2271)

^a^CBT: cognitive behavioral therapy.

^b^The *DSM-IV-TR* and *ICD-10* codes are available on request.

^c^Obsessive compulsive disorder, posttraumatic stress disorder, alcohol/drug abuse, somatoform disorders.

^d^Anxiety disorders: social phobia, panic with or without agoraphobia, agoraphobia, generalized anxiety disorder.

^e^MDD: major depressive disorder.

^f^IDS-SR: Inventory of Depressive Symptomatology, self-report version.

^g^TiC-P: Trimbos/iMTA questionnaire for Costs associated with Psychiatric Illness.

### Study Dropout

Overall, the MINI-Plus diagnostic interview could be administered to 85 of 102 patients (83%) at 10 weeks, 84 patients (82%) at 20 weeks, and 76 patients (75%) at 30 weeks. The online self-report questionnaires were completed by 77 patients at 10 weeks (76%), 65 patients (64%) at 20 weeks, and 65 patients (64%) at 30 weeks.

Patients with missing data at one or more follow-up assessments (n=49) were on average five years younger than patients who completed all assessments (n=54; mean 36.0, SD 10.5 years versus mean 41.2, SD 10.8 years; *t*_100_=2.49, *P*=.02). Within the blended CBT group, missing data were not associated with patient characteristics. Patients with missing data in the standard CBT group were more often unemployed than patients who did not have missing data (χ*^2^*_2_=14.1, *P*<.001). Figure 1 displays study and treatment adherence for both groups throughout the study period.

### Treatment Adherence

Of 102 patients, 97 (95%) started treatment and received at least one session. In blended CBT, the average number of sessions matched the planned number of 19.5 sessions (SD 8.3, range 0 to 31). On average, blended CBT had a mean of 9.6 online sessions (SD 4.4, range 0 to 16) and 10.0 face-to-face sessions (SD 4.6, range 0 to 16). Per-patient online feedback was provided a mean of 8.4 times (SD 4.2, range 0 to 15). Based on the cutoff of 14 sessions (75% of the optimal blended CBT protocol of 19 sessions), 43 of 53 blended CBT patients (81%) were considered treatment completers. When the 75% completion criterion was applied to online and face-to-face sessions separately (>7 each), 40 blended CBT patients (75%) were considered completers. Seven patients (13%) completed the full blended CBT protocol within 10 weeks.

In standard CBT, the mean number of sessions was 13.3 (SD 6.3, range 0 to 27), which was less than planned (15-18 sessions) within the time frame of this study. Based on the cutoff of 14 sessions, 22 of 49 patients (45%) were considered treatment completers. Four patients (8%) completed the standard CBT protocol (16-20 sessions) within 20 weeks.

Compared with patients in the standard CBT group, blended CBT patients received significantly more sessions in total (19 versus 13, *t*_100_=−4.09, *P*<.001), but significantly fewer face-to-face sessions at the clinic (10 versus 13, *t*_100_=3.07, *P*=.003). As expected based on the treatment protocol, mean treatment duration was significantly shorter in blended CBT than in standard CBT, with an average of 19.0 (SD 12.6) weeks versus 33.2 (SD 23.0) weeks (*t*_100_=3.91, *P*<.001). When therapist time spent on online feedback was included, the combined amount of direct and indirect therapist time did not differ between groups with an average of 14.0 (SD 6.2) hours for blended CBT and 13.3 (SD 6.3) hours for standard CBT (*t*_100_=−0.55, *P*=.58).

### Clinical Outcomes

Deviation between the desired time points and actual time of data collection was deemed within an acceptable range to enter time as a fixed categorical variable (baseline or 0 weeks, and 10, 20, and 30 weeks after start of treatment) in the linear mixed models (see Multimedia Appendix 2). No differences were found between groups in psychotropic medication use at all assessment periods. Controlling for demographic variables associated with missing data (age and employment status) did not improve model fit in all models (*P*>.05). Controlling for baseline scores did significantly improve model fit in all models with continuous outcomes (*P*<.001). Unadjusted (observed) means and outcomes per follow-up assessment are presented in Table 2. Controlling for baseline severity, no group difference was found in decrease of depression severity over time on the IDS-SR_30_ (overall: *b*=2.03, 95% CI −1.57 to 5.64; *t*_50.45_=1.17, *P*=.25). Time significantly predicted a decrease of depression severity at all time points (overall: *b*=−7.13, 95% CI −9.64 to −4.71; *t*_49.55_=−5.74, *P*<.001). For the full sample, the estimated mean depression severity decreased from severe (43.23, 95% CI 42.07-44.40) to moderate (27.1, 95% CI 22.03-32.14) [45,48].

Controlling for baseline utility scores, time significantly predicted an increase in QALYs gained at all time points (overall, *b*=.12, 95% CI 0.10-0.13; *t*_73.84_= 13.77, *P*<.001). There was no significant difference in QALY gains between groups (*b*=−0.01, 95% CI −0.03 to 0.01; *t*_73.74_=−0.88, *P*=.38).

For reliable change in depression severity (treatment response), a significant association was found between time and treatment response (*b*=1.81, OR 6.09, 95% CI 0.89-2.89; *z*=4.00, *P*<.001), but no significant difference between groups (*b*=−0.31, OR 0.73, 95% CI −1.61 to 0.99; *z* =−0.53, *P*=.60). Twenty patients reported significant improvement at all follow-up assessments (blended CBT: n=10; standard CBT: n=10). There were no patients who consecutively reported deterioration at all follow-up assessments. No deaths occurred during the study. One depression-related, but not treatment-related, serious adverse event occurred in the standard CBT group, in which one person self-harmed. Table 3 provides an overview of reliable change compared with baseline per follow-up, per group.

The proportion of patients who did not fulfill criteria for a depressive episode at 10, 20, and 30 weeks after the start of treatment did not differ significantly between groups (overall: *b*=.64, OR 1.89, 95% CI 0.73-4.90; *z*=1.31, *P*=.19). Odds of a depressive episode decreased significantly over time (overall: *b*=−1.44, OR 0.23, 95% CI 0.16-0.35; *z*=−7.12, *P*<.001). At 30-week follow-up, 23 of 102 patients (31% of full sample) met criteria for remission, reporting an absence of symptoms for at least 8 weeks. No significant between-group differences were found in the number of comorbid diagnoses. Table 4 provides an overview of the current diagnoses at 30-week follow-up for both groups.

**Table 2:** Unadjusted (observed) means and primary clinical and utility outcomes.

Outcome		Blended CBT^a^		Standard CBT		Full sample		Blended CBT vs standard CBT
		n	Value	n	Value	n	Value	*b* (95% CI)
**Depression severity (IDS-SR^b^), mean (SD)**								
	Baseline	53	45.2 (12.1)	49	41.5 (11.6)	102	43.4 (12.0)	Ref
	10 weeks	41	31.9 (12.7)	35	32.0 (17.5)	76	32.0 (15.0)	−3.36 (−8.20, 1.16)
	20 weeks	35	30.7 (16.1)	30	27.1 (15.7)	65	29.0 (15.9)	1.50 (−6.22, 9.02)
	30 weeks	36	29.5 (17.2)	29	21.1 (15.4)	65	25.8 (16.9)	8.92 (−1.05, 18.50)
**QALYs^c^ (EQ-5D-3L), mean (SD)**								
	Baseline	53	0	49	0	102	0	Ref
	10 weeks	41	0.09 (0.05)	35	0.10 (0.05)	76	0.10 (0.05)	−0.01 (−0.04, 0.01)
	20 weeks	35	0.20 (0.10)	30	0.24 (0.09)	65	0.22 (0.10)	−0.02 (−0.06, 0.03)
	30 weeks	36	0.31 (0.16)	29	0.39 (0.13)	65	0.35 (0.15)	−0.03 (−0.10, 0.04)
**Reliable change (IDS-SR), n (%)**								
	Baseline	53	0	49	0	102	0	Ref
	10 weeks	41	27 (66)	35	19 (54)	76	46 (61)	0.89 (−1.17, 2.72)
	20 weeks	35	21 (60)	30	19 (63)	65	40 (62)	0.07 (−1.69, 2.01)
	30 weeks	36	22 (61)	29	23 (79)	65	45 (69)	−0.48 (−2.49, 1.47)
**Current depressive episode (MINI-Plus)^d^, n (%)**								
	Baseline	53	52 (98)	49	48 (98)	102	100 (98)	Ref
	10 weeks	44	27 (61)	41	18 (44)	85	45 (44)	1.44 (−2.19, 5.07)
	20 weeks	44	24 (55)	40	15 (38)	84	39 (38)	1.30 (−2.35, 5.00)
	30 weeks	39	15 (39)	37	13 (35)	76	28 (28)	0.32 (−3.35, 4.00)

^a^CBT: cognitive behavioral therapy.

^b^IDS-SR: Inventory of Depressive Symptomatology, self-report version.

^c^MINI-Plus: Mini-International Neuropsychiatric Interview Plus.

^d^QALYs: quality-adjusted life-years.

**Table 3:** Reliable change in depression severity.

Change at each follow-up		Blended CBT^a^	Standard CBT	Full sample
**Week 10**		**n=41**	**n=35**	**n=76**
	Deterioration, n (%)	1 (2)	3 (9)	4 (5)
	No change, n (%)	16 (39)	16 (48)	32 (42)
	Improvement, n (%)	21 (68)	10 (29)	31 (41)
	Remission, n (%)	3 (7)	6 (17)	9 (12)
**Week 20**		**n=35**	**n=30**	**n=65**
	Deterioration, n (%)	0 (0)	0 (0)	0 (0)
	No change, n (%)	16 (46)	13 (43)	29 (45)
	Improvement, n (%)	13 (37)	11 (37)	24 (37)
	Remission, n (%)	6 (17)	6 (20)	12 (19)
**Week 30**		**n=36**	**n=29**	**n=65**
	Deterioration, n (%)	1 (3)	0 (0)	1 (2)
	No change, n (%)	15 (42)	10 (35)	25 (39)
	Improvement, n (%)	14 (40)	8 (23)	22 (34)
	Remission, n (%)	6 (17)	11 (38)	17 (26)

^a^ CBT: cognitive behavioral therapy.

**Table 4:** Current DSM-IV-TR^a^ diagnoses at 30-week follow-up.

Diagnosis	Blended CBT^b^ (n=39)	Standard CBT (n=37)	Full sample (N=76)
Any comorbidity, n (%)	17 (44)	18 (49)	35 (46)
Depressive episode, n (%)	15 (39)	13 (27)	28 (37)
Anxiety, n (%)	15 (28)	15 (31)	30 (29)
OCD^c^, n (%)	6 (11)	3 (6)	9 (9)
PTSD^d^, n (%)	3 (6)	2 (4)	5 (5)
Somatoform, n (%)	4 (8)	4 (8)	8 (8)
Alcohol/drug dependency, n (%)	1 (2)	3 (6)	4 (4)
Comorbid disorders, mean (SD)	1.5 (2.0)	1.3 (1.7)	1.4 (1.9)

^a^DSM-IV: *Diagnostic and Statistical Manual of Mental Disorders* (Fourth Edition).

^b^CBT: Cognitive behavioral therapy.

^c^OCD: obsessive compulsive disorder.

^d^PTSD: posttraumatic stress disorder.

### Costs

Before examining cost-effectiveness, costs were explored in separate linear mixed models, controlling for baseline costs. Patients in the blended CBT group reported higher cumulative societal costs on average than patients in standard CBT (overall: *b*=1410, 95% CI −28.5 to 2776.7; *t*_78.86_=2.06, *P*=.04). Part of this effect appeared to be driven by rare and costly medical events. When costs of hospitalization and in-patient psychiatric care were not included in the societal costs in a sensitivity analysis, the between-group difference was reduced (overall: *b*=1206, 95% CI −300.9 to 2713.3; *t*_76.99_=1.57, *P*=.12). Based on the estimated marginal means from the mixed-effects model, the overall societal costs for the full sample at 30 weeks were estimated to be mean €10,075 (95% CI €8086-€12,066).

Regarding average cumulative direct medical costs, both groups reported similar costs (overall: *b*=55, 95% CI −477.0 to 576.5; *t*_90.19_=0.22, *P*=.83). Average direct medical costs at 30 weeks were estimated to be €4535 (95% CI €3789-€5282). Sensitivity analyses showed that for cumulative indirect nonmedical costs (costs associated with absenteeism and presenteeism), patients in the blended CBT group on average reported higher costs compared with standard CBT (overall: *b*=140, 95% CI −178.4 to 448.4; *t*_76.22_=0.89, *P*=.37). Average indirect nonmedical costs at 30 weeks were estimated to be €1413 (95% CI €954-€1872). Unadjusted (observed) means and outcomes per follow-up assessment are presented in Table 5.

### Cost-Effectiveness and Cost Utility

The results from the cost-effectiveness and cost-utility analyses are presented in Table 6. Estimated between-group mean differences in costs, utility, and effects over the full study period were not significant.

**Table 5:** Unadjusted mean cumulative costs in Euros.

Costs		Blended CBT		Standard CBT		Full sample		Blended CBT vs standard CBT
		n	Mean (SD)	n	Mean (SD)	n	Mean (SD)	*b* (95% CI)
**Societal costs (€)**								
	Baseline	53	2205 (2615)	49	1401 (1761)	102	1819 (891)	Ref
	10 weeks	40	5140 (5229)	35	2996 (2608)	75	4140 (2419)	1482 (−114, 2989)
	20 weeks	33	9523 (9466)	28	5765 (4974)	61	7798 (5202)	3137 (288, 5980)
	30 weeks	30	12,401 (13,198)	24	8493 (7239)	54	10,664 (6316)	3923 (−148, 7963)
**Direct medical costs (€)**								
	Baseline	53	628 (888)	49	411 (389)	102	524 (381)	Ref
	10 weeks	40	2363 (2743)	35	1697 (934)	75	2052 (1661)	322 (−257, 920)
	20 weeks	33	3834 (3833)	28	3025 (1846)	61	3463 (2610)	308 (−734, 1343)
	30 weeks	30	4728 (4504)	24	4.287 (3743)	54	4527 (3398)	25 (−1525, 1512)

^a^CBT: Cognitive behavioral therapy.

**Table 6:** Results of the cost-effectiveness and cost-utility analyses.

Outcome		Blended CBT^a^ vs standard CBT		ICER^b^	Cost-effectiveness plane distribution^c^ (%)			
		Difference cost, mean difference (95% CI)	Difference effect, mean or risk difference^d^ (95% CI)		NW^e^	SW^f^	NE^g^	SE^h^
**Societal perspective**								
	RCI^i^	1183 (−399, 2765)	0.03 (−.010, .15)	39,433	10.0	—	89.2	0.7
	MINI^j^	1183 (−399, 2765)	0.06 (−0.05, 0.19)	19,716	89.2	0.7	10.0	—
	QALY^k^	1183 (−399, 2765)	0.01 (−0.03, 0.04)	185,880	34.4	0.6	64.9	0.2
**Health care provider perspective**								
	RCI	−176 (−659, 343)	0.03 (−0.10, 0.15)	−5867	5.2	4.9	20.2	69.7
	MINI	−176 (−659, 343)	0.06 (−0.05, 0.19)	−2933	20.2	69.7	5.2	4.9
	QALY	−176 (−659, 343)	0.01 (−0.03, 0.04)	−29,333	3.0	31.9	22.3	42.7

^a^CBT: Cognitive behavioral therapy.

^b^ICER: incremental cost-effectiveness ratio.

^c^For plane distribution, NW=more expensive, less effective; SW=less expensive, less effective; NE=more expensive, more effective; SE=less expensive, more effective.

^d^Risk difference: RCI and MINI; Mean: QALY.

^e^NW: northwest.

^f^SW: southwest.

^g^NE: northeast.

^h^SE: southeast.

^i^RCI: reliable change index (based on IDS-SR).

^j^MINI: Mini-International Neuropsychiatric Interview Plus (diagnostic interview diagnosis of a depressive episode).

^k^QALY: quality-adjusted life-year (based on EQ-5D-3L).

#### Societal Perspective

**Figure 2:**
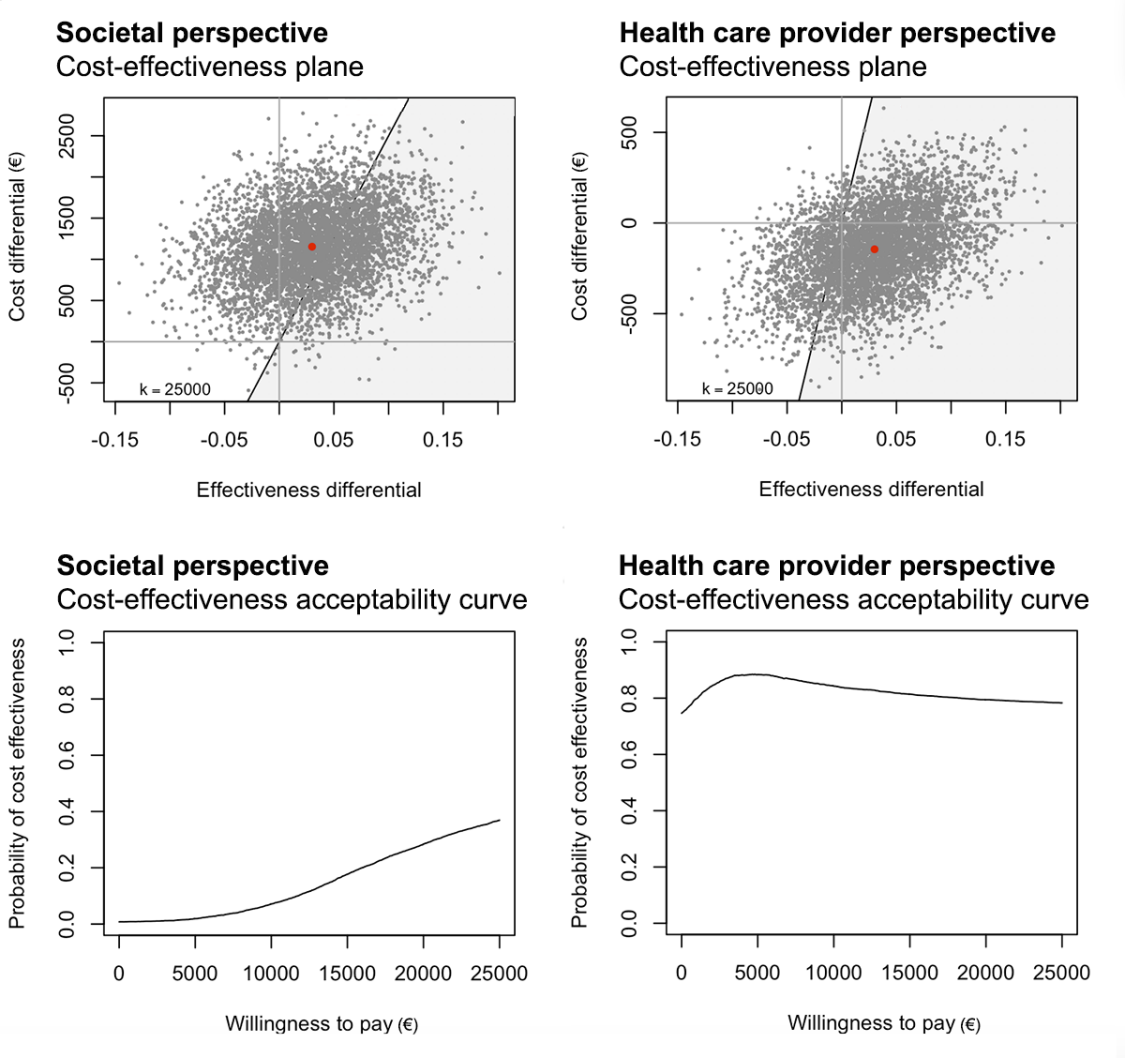
Cost-effectiveness planes for response to treatment (blended CBT versus standard CBT) from the societal perspective (top left) and health care provider perspective (top right), and cost-effectiveness acceptability curves for response to treatment (blended CBT versus standard CBT) from the societal perspective (bottom left) and health care provider perspective (bottom right). CBT: cognitive behavioral therapy

For response to treatment, the ICER was 39,433, meaning that an additional treatment response in blended CBT was associated with €39,433 higher costs compared with standard CBT. Of the estimated cost-effect pairs, 89.2% was located in the northeast quadrant (more effective and more expensive), 10% in the northwest quadrant (less effective and more expensive), and 0.7% in the southeast quadrant (more effective and less expensive). The probability of blended CBT being cost-effective compared with standard CBT was 0.01 at a ceiling ratio of €0 per additional response to treatment, and 0.02, 0.18, and 0.28 at ceiling ratios of €5000, €15,000, and €20,000 per response to treatment, respectively. The probability of blended CBT being cost-effective compared with standard CBT rose to 0.37 at a ceiling ratio of €25,000 per additional response to treatment. The cost-effectiveness plane and cost-effectiveness acceptability curve are presented in Figure 2.

For occurrence of depressive episodes, the ICER was 19,716. This means that avoiding an additional depressive episode in blended CBT was associated with €19,716 higher costs compared with standard CBT. The probability of blended CBT being cost-effective compared with standard CBT was 0.01 at a ceiling ratio of €0 per depressive episode. This was the highest possible probability.

For QALYs, the ICER was 185,880, meaning that one extra QALY in blended CBT was associated with €185,880 higher costs compared with standard CBT. The probability of blended CBT being cost-effective compared with standard CBT was 0.01 at a ceiling ratio of €0 per QALY. The probability of blended CBT being cost-effective compared with standard CBT rose to 0.02 at a ceiling ratio of €25,000 per extra QALY.

#### Health Care Provider Perspective

For response to treatment, the ICER was −5867, meaning that an additional treatment response in blended CBT was associated with €5867 lower costs compared with standard CBT. Of the estimated cost-effect pairs, 69.7% was located in the southeast quadrant (more effective and less expensive), 20.2% in the northeast quadrant (more effective and more expensive), 5.2% in the northwest quadrant (less effective and more expensive), and 4.9% in the southwest quadrant (less effective, less expensive). The probability of blended CBT being cost-effective compared with standard CBT was 0.75 at a ceiling ratio of €0 per additional response to treatment, and 0.80 at a ceiling ratio of €1000 per additional response to treatment. The probability of blended CBT being cost-effective compared with standard CBT rose to 0.88 at a ceiling ratio of €5000 per additional response to treatment. The cost-effectiveness plane and cost-effectiveness acceptability curve are presented in Figure 2.

For occurrence of depressive episodes, the ICER was −2933. This means that an additional depressive episode in blended CBT was associated with €2933 lower costs compared with standard CBT. The probability of blended CBT being cost-effective compared with standard CBT was 0.75 at a ceiling ratio of €0 per depressive episode.

For QALYs, the ICER was −29,333, meaning that one extra QALY in blended CBT was associated with €29,333 lower costs compared with standard CBT. The probability of blended CBT being cost-effective compared with standard CBT was 0.75 at a ceiling ratio of €0 per QALY gained, and 0.81 at €5000 per QALY gained. The probability of blended CBT being cost-effective compared with standard CBT rose to 0.85 at €10,000 per QALY gained.

## Discussion

### Overview

This pilot RCT focused on depression treatment in Dutch routine specialized mental health care and compared the costs and effects of blended CBT for MDD to standard (evidence-based) face-to-face CBT over a 30-week time frame. The study examined whether blended CBT has the potential to lead to clinical effects that are comparable to the effects of standard CBT, at lower costs. To the best of our knowledge, our study was the first to explore the cost-effectiveness of integrated (rather than add-on) blended depression treatment in routine mental health care and compare this blended treatment to existing, evidence-based, face-to-face CBT.

### Principal Findings

The results suggest that blended CBT for depression has the potential to lead to costs and clinical effects that are comparable to the costs and effects associated with standard CBT. In both treatment groups, the severity of depressive symptoms decreased, the probability of having a depression diagnosis lessened, and quality of life improved. When costs and effects were combined, results were mixed. From a societal perspective, which includes productivity losses, blended CBT was not cost-effective compared with standard CBT. From a health care provider perspective, blended CBT had an acceptable probability of being cost-effective compared with standard CBT for treatment response and QALYs (cost utility), but not for depression diagnosis (depressive episodes).

Results show that blended CBT patients received significantly fewer face-to-face sessions than standard CBT patients (10 versus 13 sessions). In line with our expectations, blended CBT also led to shorter treatment duration (19 versus 33 weeks). After 20 weeks of treatment, 51% (27 of 53) of blended CBT patients completed 75% or more of the treatment protocol (at least 14 of 18 sessions in total) versus 5% in the CBT group (3 of 49). After 30 weeks, the percentage of treatment completers rose to 85% for blended CBT and 45% for standard CBT. Combined with therapist time required for online feedback, blended CBT and standard CBT unexpectedly required similar per-patient time investments from therapists (14 versus 13 hours). This was mainly driven by the mean number of sessions in standard CBT, which was considerably lower than expected based on the protocol (13 versus 18 sessions).

### Comparison with Other Work

Because this is one of the first studies to compare both costs and effects of blended depression treatment to treatment as usual, the possibility of comparing our findings to those of previous research is limited. The clinical findings appear to be in line with other studies that examined the clinical effectiveness of combined online and face-to-face treatment for depression. However, in this study we defined blended treatment as an integrated approach, replacing face-to-face sessions with online modules rather than a combined approach that adds an online intervention to a face-to-face CBT protocol. For example, Berger and colleagues [33] evaluated the clinical effectiveness of an evidence-based unguided CBT self-help intervention (Deprexis) combined with regular psychotherapy in outpatient specialized depression care compared with regular psychotherapy. The results of their RCT (N=98) showed superiority of the combined treatment [33] over regular treatment for reduced depressive symptoms (via Beck Depression Inventory-II, BDI-II) (Cohen *d*=.51) at posttreatment (12 weeks). In another RCT (N=229), this unguided self-help intervention was added to a psychodynamic psychotherapeutic treatment (treatment as usual) for depressed inpatients and compared with online information plus treatment as usual [35]. After treatment, treatment as usual plus unguided self-help was superior to treatment as usual plus active control (Cohen *d*=.44).

A naturalistic study in routine specialized mental health care by Kenter and colleagues [36] showed comparable clinical effects for blended and standard face-to-face treatment for depressed patients, as measured with Global Assessment of Functioning (GAF) scores (N=3175) [36]. In this study, the combined rather than integrated approach led to longer treatment duration and more treatment sessions (combined total of online and face-to-face sessions), making blended treatment by design more costly than face-to-face treatment [36].

Offering blended treatment in a group format rather than to patients individually could potentially further lower costs associated with treatment. Schuster and colleagues [64] examined change in severity of depressive symptoms (via Center for Epidemiologic Studies Depression Scale, CES-D) in a combined blended group therapy compared with a waitlist control group. Participants (N=46) were adults with depressive symptoms who were recruited from the general population in Austria. Blended therapy consisted of eight 90-minute group sessions, combined with access to an e-learning platform. Treatment was provided at the University of Salzburg. The group intervention was superior to waitlist (Cohen *d*=.87) after treatment.

Finally, in a recent study by Thase and colleagues [25], an integrated approach was chosen for treating medication-free adults with MDD (N=154). Treatment was provided in two university clinics. In the blended treatment, nine online modules were combined with 12 face-to-face sessions of 25 minutes. Compared with 20 CBT sessions of 50 minutes, blended CBT led to noninferior results (Cohen *d*=.05) on the HAM-D after 16 weeks of treatment.

### Limitations

There are a number of factors that need to be considered when interpreting the findings. First, the sample size was smaller than anticipated, which led to less stable probability estimates. This was mainly due to time constraints of conducting the study. The achieved sample is considered adequate for the central aim of the study, which was to guide further development and research in blended CBT. Future studies should focus on hypothesis testing in larger samples, using a noninferiority design.

Second, within this study, patients could be followed for 30 weeks. Although this was considered an acceptable time period to assess changes in outcomes during and shortly after treatment, future studies could include a long-term follow-up (eg, 1 year after treatment). This would provide more insight into the course of depression, treatment trajectories, quality of life, and costs over time. When examining costs, future studies could also include specific costs associated with delivering the interventions, such as the hosting and maintenance of the online treatment platform. Unfortunately, these data were not available in this study.

Third, this sample was characterized by a high level of heterogeneity, both in clinical and cost characteristics. On the one hand, this is a positive aspect because it is representative of the population in routine specialized mental health care. On the other hand, it led to some baseline imbalances between groups. This is most apparent in the average direct medical and nonmedical costs patients reported in the month before baseline, which were higher in the blended CBT group than the standard CBT group. This was primarily caused by the fact that three patients in the blended group reported having been admitted to an in-patient psychiatric ward before baseline assessment versus no patients in the standard CBT group.

The imbalances at baseline increased the uncertainty around the cost and effectiveness estimates, especially at the 30-week follow-up. This is reflected in the analyses concerning treatment response, quality of life, and direct medical costs (health care provider perspective). The models that included time were in favor of the standard CBT group, whereas the models that did not include time were in favor of blended CBT. It is important to keep in mind that all findings were nonsignificant and varied around zero, signifying no differences between groups.

Finally, it should be noted that in the study protocol [41] more outcomes were included than could be reported on in this paper, such as working alliance and depressive cognitions. Information on these outcomes will be provided in forthcoming publications. Next, in the study protocol, it was stated that treatment response would be assessed at 30 weeks. This might lead readers to wonder whether there was a deviation from the planned analyses. However, within the cost-effectiveness framework, the focus lies on cumulative outcomes. Therefore, cumulative group differences were estimated with linear mixed models over the full study period, rather than estimating a group difference at a single time point (30 weeks).

### Conclusion

This study is one of the first to examine both costs and effects of integrated blended CBT for depression in specialized mental health care. The results are promising and suggest it is feasible to digitalize part of the therapist-patient interaction, even in the complex patient population that characterizes specialized mental health care. Blended CBT appears to lead to comparable clinical effects as standard CBT, may increase treatment adherence, and could potentially speed up patient flow through the treatment process. From a societal perspective, blended CBT is not considered cost-effective compared with standard CBT. However, there is an acceptable probability that blended CBT is cost-effective from the perspective of the health care provider. Further research in a larger sample seems warranted, which should focus on optimizing the clinical effects of blended treatment as well as the cost-impact.

## Appendix

Multimedia Appendix 1 Cost categories and prices (€, 2014).

Multimedia Appendix 2 Compliance with questionnaires at follow-up.

Multimedia Appendix 3 CONSORT-EHEALTH checklist (V 1.6.1).

## Abbreviations

CBT: cognitive behavioral therapy
ICER: Incremental Cost-Effectiveness Ratio
IDS-SR: Inventory of Depressive Symptomatology Self-Report
MDD: major depressive disorder
MINI-Plus: Mini-International Neuropsychiatric Interview Plus
QALY: quality-adjusted life-year
RCI: reliable change index
TiC-P: Trimbos and iMTA questionnaire on Costs associated with Psychiatric illness

## References

[1] Cuijpers P, Huibers M, Ebert DD, Koole SL, Andersson G (2013). How much psychotherapy is needed to treat depression? A metaregression analysis. J Affect Disord.

[2] Karyotaki E, Smit Y, Holdt Henningsen K, Huibers MJ, Robays J, de Beurs D, Cuijpers P (2016). Combining pharmacotherapy and psychotherapy or monotherapy for major depression? A meta-analysis on the long-term effects. J Affect Disord.

[3] Butler AC, Chapman JE, Forman EM, Beck AT (2006). The empirical status of cognitive-behavioral therapy: a review of meta-analyses. Clin Psychol Rev.

[4] Kohn R, Saxena S, Levav I, Saraceno B (2004). The treatment gap in mental health care. Bull World Health Organ.

[5] Bremmer F, van Es M (2013). Een analyse van de verwachte kosten en baten van eHealth-blended behandelen en begeleiden.

[6] Araya R, Zitko P, Markkula N, Rai D, Jones K (2018). Determinants of access to health care for depression in 49 countries: a multilevel analysis. J Affect Disord.

[7] Health Quality Ontario (2017). Psychotherapy for major depressive disorder and generalized anxiety disorder: a health technology assessment. Ont Health Technol Assess Ser.

[8] Andersson G (2010). The promise and pitfalls of the internet for cognitive behavioral therapy. BMC Med.

[9] Ruwaard J, Kok RN (2015). Wild West eHealth: Time to hold our horses?. Eur Health Psychol.

[10] Emmelkamp PM, David D, Beckers T, Muris P, Cuijpers P, Lutz W, Andersson G, Araya R, Banos Rivera RM, Barkham M, Berking M, Berger T, Botella C, Carlbring P, Colom F, Essau C, Hermans D, Hofmann SG, Knappe S, Ollendick TH, Raes F, Rief W, Riper H, van der Oord S, Vervliet B (2014). Advancing psychotherapy and evidence-based psychological interventions. Int J Methods Psychiatr Res.

[11] Andrews G, Basu A, Cuijpers P, Craske M, McEvoy P, English C, Newby J (2018). Computer therapy for the anxiety and depression disorders is effective, acceptable and practical health care: An updated meta-analysis. J Anxiety Disord.

[12] Wright JH, Wright AS, Albano AM, Basco MR, Goldsmith LJ, Raffield T, Otto MW (2005). Computer-assisted cognitive therapy for depression: maintaining efficacy while reducing therapist time. Am J Psychiatry.

[13] Richards D, Richardson T (2012). Computer-based psychological treatments for depression: a systematic review and meta-analysis. Clin Psychol Rev.

[14] Andersson G, Cuijpers P (2009). Internet-based and other computerized psychological treatments for adult depression: a meta-analysis. Cogn Behav Ther.

[15] Andersson G, Rozental A, Shafran R, Carlbring P (2018). Long-term effects of internet-supported cognitive behaviour therapy. Expert Rev Neurother.

[16] Webb CA, Rosso IM, Rauch SL (2017). Internet-based cognitive-behavioral therapy for depression: current progress and future directions. Harv Rev Psychiatry.

[17] Carlbring P, Andersson G, Cuijpers P, Riper H, Hedman-Lagerlöf E (2018). Internet-based vs face-to-face cognitive behavior therapy for psychiatric and somatic disorders: An updated systematic review and meta-analysis. Cogn Behav Ther.

[18] Kolovos S, van Dongen JM, Riper H, Buntrock C, Cuijpers P, Ebert DD, Geraedts AS, Kenter RM, Nobis S, Smith A, Warmerdam L, Hayden JA, van Tulder MW, Bosmans JE (2018). Cost effectiveness of guided Internet-based interventions for depression in comparison with control conditions: an individual-participant data meta-analysis. Depress Anxiety.

[19] Cuijpers P, Riper H, Andersson G (2015). Internet-based treatment of depression. Curr Opin Psychol.

[20] Schueller SM, Tomasino KN, Mohr DC (2016). Integrating human support into behavioral intervention technologies: the efficiency model of support. Clin Psychol Sci Pract.

[21] Wentzel J, van der Vaart R, Bohlmeijer ET, van Gemert-Pijnen JE (2016). Mixing online and face-to-face therapy: how to benefit from blended care in mental health care. JMIR Ment Health.

[22] Kooistra LC, Ruwaard J, Wiersma JE, van Oppen P, van der Vaart R, van Gemert-Pijnen JE, Riper H (2016). Development and initial evaluation of blended cognitive behavioural treatment for major depression in routine specialized mental health care. Internet Interv.

[23] Erbe D, Eichert HC, Riper H, Ebert DD (2017). Blending face-to-face and Internet-based interventions for the treatment of mental disorders in adults: systematic review. J Med Internet Res.

[24] Andersson G, Cuijpers P (2008). Pros and cons of online cognitive-behavioural therapy. Br J Psychiatry.

[25] Thase ME, Wright JH, Eells TD, Barrett MS, Wisniewski SR, Balasubramani GK, McCrone P, Brown GK (2018). Improving the efficiency of psychotherapy for depression: computer-assisted versus standard CBT. Am J Psychiatry.

[26] Kleiboer A, Smit J, Bosmans J, Ruwaard J, Andersson G, Topooco N, Berger T, Krieger T, Botella C, Baños R, Chevreul K, Araya R, Cerga-Pashoja A, Cieślak R, Rogala A, Vis C, Draisma S, van Schaik DJ, Kemmeren L, Ebert D, Berking M, Funk B, Cuijpers P, Riper H (2016). European COMPARative Effectiveness research on blended depression treatment versus treatment-as-usual (E-COMPARED): study protocol for a randomized controlled, non-inferiority trial in eight European countries. Trials.

[27] Romijn G, Riper H, Kok R, Donker T, Goorden M, Hakkaart-van Roijen L, Kooistra LC, van Balkom A, Koning J (2015). Cost-effectiveness of blended vs face-to-face cognitive behavioural therapy for severe anxiety disorders: study protocol of a randomized controlled trial. BMC Psychiatry.

[28] Vis C, Kleiboer A, Prior R, Bønes E, Cavallo M, Clark S, Dozeman E, Ebert D, Etzelmueller A, Favaretto G, Zabala Af, Kolstrup N, Mancin S, Mathiassen K, Myrbakk VN, Mol M, Jimenez JP, Power K, van Schaik DJF, Wright C, Zanalda E, Pederson CD, Smit J, Riper H (2015). Implementing and up-scaling evidence-based eMental health in Europe: the study protocol for the MasterMind project. Internet Interventions.

[29] Mol M, Dozeman E, van Schaik DJ, Vis CP, Riper H, Smit JH (2016). The therapist's role in the implementation of internet-based cognitive behavioural therapy for patients with depression: study protocol. BMC Psychiatry.

[30] Hickie IB, Davenport TA, Luscombe GM, Moore M, Griffiths KM, Christensen H (2010). Practitioner-supported delivery of internet-based cognitive behaviour therapy: evaluation of the feasibility of conducting a cluster randomised trial. Med J Aust.

[31] Høifødt RS, Lillevoll KR, Griffiths KM, Wilsgaard T, Eisemann M, Waterloo K, Kolstrup N (2013). The clinical effectiveness of web-based cognitive behavioral therapy with face-to-face therapist support for depressed primary care patients: randomized controlled trial. J Med Internet Res.

[32] Kessler D, Lewis G, Kaur S, Wiles N, King M, Weich S, Sharp DJ, Araya R, Hollinghurst S, Peters TJ (2009). Therapist-delivered Internet psychotherapy for depression in primary care: a randomised controlled trial. Lancet.

[33] Berger T, Krieger T, Sude K, Meyer B, Maercker A (2018). Evaluating an e-mental health program. J Affect Disord.

[34] Meyer B, Berger T, Caspar F, Beevers CG, Andersson G, Weiss M (2009). Effectiveness of a novel integrative online treatment for depression (Deprexis): randomized controlled trial. J Med Internet Res.

[35] Zwerenz R, Becker J, Knickenberg RJ, Siepmann M, Hagen K, Beutel ME (2017). Online self-help as an add-on to inpatient psychotherapy: efficacy of a new blended treatment approach. Psychother Psychosom.

[36] Kenter RM, van de Ven PM, Cuijpers P, Koole G, Niamat S, Gerrits RS, Willems M, van Straten A (2015). Costs and effects of Internet cognitive behavioral treatment blended with face-to-face treatment: results from a naturalistic study. Internet Interventions.

[37] American Psychiatric Association (2000). Diagnostic and Statistical Manual of Mental Disorders, 4th Edition, Text Revision (DSM-IV-TR).

[38] Spijker J, Bockting CL, Meeuwissen JA, van Vliet IM, Emmelkamp PM, Hermens ML, van Balkom AJL (2013). Multidisciplinaire richtlijn depressie (derde revisie). Richtlijn voor de diagnostiek, behandeling en begeleiding van volwassen patiënten met een depressieve stoornis.

[39] Sheehan DV, Lecrubier Y, Sheehan KH, Amorim P, Janavs J, Weiller E, Hergueta T, Baker R, Dunbar GC (1998). The Mini-International Neuropsychiatric Interview (M.I.N.I.): the development and validation of a structured diagnostic psychiatric interview for DSM-IV and ICD-10. J Clin Psychiatry.

[40] van Vliet IM, de Beurs E (2007). Het Mini Internationaal Neuropsychiatrisch Interview (MINI): een kort gestructureerd diagnostisch psychiatrisch interview voor DSM-IV en ICD-10-stoornissen. Tijdschrift voor de Psychiatrie.

[41] Kooistra LC, Wiersma JE, Ruwaard J, van Oppen P, Smit F, Lokkerbol J, Cuijpers P, Riper H (2014). Blended vs. face-to-face cognitive behavioural treatment for major depression in specialized mental health care: study protocol of a randomized controlled cost-effectiveness trial. BMC Psychiatry.

[42] Bockting CL, Huibers MJ, Keijsers GPJ GJ, van Minnen A, Hoogduin CA (2011). Protocollaire behandeling van patienten met een depressieve stoornis. Protocollaire behandelingen voor volwassenen met psychische klachten deel 1.

[43] Beck JS (2011). Cognitive Behavior Therapy: Basics and Beyond.

[44] Beck AT, Rush AJ, Shaw BF, Emery G (1980). Cognitive Therapy of Depression.

[45] Rush AJ, Trivedi MH, Ibrahim HM, Carmody TJ, Arnow B, Klein DN, Markowitz JC, Ninan PT, Kornstein S, Manber R, Thase ME, Kocsis JH, Keller MB (2003). The 16-Item Quick Inventory of Depressive Symptomatology (QIDS), clinician rating (QIDS-C), and self-report (QIDS-SR): a psychometric evaluation in patients with chronic major depression. Biol Psychiatry.

[46] Rush AJ, Carmody T, Reimitz P (2000). The Inventory of Depressive Symptomatology (IDS): clinician (IDS‐C) and self‐report (IDS‐SR) ratings of depressive symptoms. Int J Method Psychiat Res.

[47] Cusin C, Yang H, Yeung A, Fava M, Bear L, Blais MA (2019). Rating scales for depression. Handbook of Clinical Rating Scales and Assessment in Psychiatry and Mental Health.

[48] Rush AJ, Gullion CM, Basco MR, Jarrett RB, Trivedi MH (1996). The Inventory of Depressive Symptomatology (IDS): psychometric properties. Psychol Med.

[49] Trivedi MH, Rush AJ, Ibrahim HM, Carmody TJ, Biggs MM, Suppes T, Crismon ML, Shores-Wilson K, Toprac MG, Dennehy EB, Witte B, Kashner TM (2004). The Inventory of Depressive Symptomatology, Clinician Rating (IDS-C) and Self-Report (IDS-SR), and the Quick Inventory of Depressive Symptomatology, Clinician Rating (QIDS-C) and Self-Report (QIDS-SR) in public sector patients with mood disorders: a psychometric evaluation. Psychol Med.

[50] Jacobson NS, Truax P, Kazdin AE (1991). Clinical significance: a statistical approach to defining meaningful change in psychotherapy research. Methodological Issues and Strategies in Clinical Research.

[51] Trivedi MH, Rush AJ, Wisniewski SR, Nierenberg AA, Warden D, Ritz L, Norquist G, Howland RH, Lebowitz B, McGrath PJ, Shores-Wilson K, Biggs MM, Balasubramani GK, Fava M, STAR*D study team (2006). Evaluation of outcomes with citalopram for depression using measurement-based care in STAR*D: implications for clinical practice. Am J Psychiatry.

[52] EuroQol Group (1990). EuroQol--a new facility for the measurement of health-related quality of life. Health Policy.

[53] Lamers LM, McDonnell J, Stalmeier PF, Krabbe PF, Busschbach JJ (2006). The Dutch tariff: results and arguments for an effective design for national EQ-5D valuation studies. Health Econ.

[54] Hakkaart-van Roijen L, van Straten A, Tiemens B, Donker M (2002). Trimbos/iMTA questionnaire for costs associated with psychiatric illness (TIC-P).

[55] Hakkaart-van Roijen L, van der Linden N, Bouwmans C, Kanters T, Tan S (2016). Kostenhandleiding: methodologie van kostenonderzoek en referentieprijzen voor economische evaluaties in de gezondheidszorg.

[56] Hakkaart-Van Roijen L, Tan SS, Bouwmans CA (2010). Handleiding voor kostenonderzoek--methoden en standaard kostprijzen voor economische evaluaties in de gezondheidszorg.

[57] (2016). Z-index.

[58] Hesser H (2015). Modeling individual differences in randomized experiments using growth models: recommendations for design, statistical analysis and reporting of results of internet interventions. Internet Interventions.

[59] White IR, Carpenter J, Horton NJ (2012). Including all individuals is not enough: lessons for intention-to-treat analysis. Clinical Trials.

[60] Bates D, Mächler M, Bolker B, Walker S (2015). Fitting linear mixed-effects models using lme4. J Stat Soft.

[61] R Core Team (2018). R: a language and environment for statistical computing.

[62] Fenwick E, O'Brien BJ, Briggs A (2004). Cost-effectiveness acceptability curves--facts, fallacies and frequently asked questions. Health Econ.

[63] Hakkaart-van Roijen L, van Straten A, Maiwenn A, Rutten F, Donker M (2006). Cost-utility of brief psychological treatment for depression and anxiety. Br J Psychiatry.

[64] Schuster R, Leitner I, Carlbring P, Laireiter A (2017). Exploring blended group interventions for depression: randomised controlled feasibility study of a blended computer- and multimedia-supported psychoeducational group intervention for adults with depressive symptoms. Internet Interv.

