# Supplementary material for: Cost and Effectiveness of Blended Versus Standard Cognitive Behavioral Therapy for Outpatients With Depression in Routine Specialized Mental Health Care: Pilot Randomized Controlled Trial
Source: J Med Internet Res. 2019 Oct 29;21(10):e14261. doi: 10.2196/14261 (PMC6914243; doi:10.2196/14261)
Supplement: Multimedia Appendix 1 [file jmir_v21i10e14261_app1.pdf]

Multimedia Appendix 1. Cost categories and prices (€, 2014).

Cost category	Unit	Unit cost price	Travel costs
Direct medical			
General (mental) health care			
General practitioner	Visit	33.00	4.29
Nurse practitioner	Visit	17.00	4.29
Family aid	Home visit	58.00	-
Nurse	Home visit	30.00	-
Dietician	Visit	29.70	3.42
Physiotherapist	Visit	33.00	3.42
Occupational health specialist	Visit	73.11	6.34
Social worker	Visit	65.00	3.42
Alternative healer	Visit	33.00	3.42
Outpatient mental health care			
Psychotherapist, psychologist or psychiatrist at an independent practice	Visit	81.00	3.95
Psychotherapist, psychologist or psychiatrist at a specialized center	Visit	112.00	4.33
Self-help group	Visit	16.00	3.32
Day-treatment	Day	169.40	4.33
Crisis department	Contact	139.67	
Inpatient mental health care			
Admission to an open psychiatric ward	Day	302.36	4.33
Admission to a closed psychiatric ward	Day	302.36	4.33
Specialized somatic health care			
Hospital emergency department	Visit	259.00	4.33
Ambulance rides	Ride	515.00	-
Direct non-medical			
Help with household or informal care	Hour	14.00	-
Indirect non-medical			

Absenteeism paid work	Hour	Male: 37.90<br>Female: 31.60	
Absenteeism unpaid work	Hour	14.00	
Presenteeism paid work	Hour	Male: 37.90<br>Female: 31.60	
Presenteeism unpaid work	Hour	14.00	
