# Supplementary material for: Cost and Effectiveness of Blended Versus Standard Cognitive Behavioral Therapy for Outpatients With Depression in Routine Specialized Mental Health Care: Pilot Randomized Controlled Trial
Source: J Med Internet Res. 2019 Oct 29;21(10):e14261. doi: 10.2196/14261 (PMC6914243; doi:10.2196/14261)
Supplement: Multimedia Appendix 2 [file jmir_v21i10e14261_app2.pdf]

Multimedia Appendix 2. Compliance with questionnaires at follow-up.[a]

Time-point	Blended CBT<br>N=53	Standard CBT<br>N=49	Total sample<br>N=102
T1 (10 weeks)	n=41	n=35	n=76
Mean (SD)	11.4 (2.0)	10.6 (1.2)	11.0 (1.7)
Median, range	10, 10-19	10, 10-15	10, 10-19
Quartiles (25, 50, 75)	10, 10, 12	10, 10, 11	10, 10, 11
T2 (20 weeks)	n=35	n=30	n=65
Mean (SD)	20.9 (1.5)	21.0 (1.7)	20.9 (1.6)
Median, range	20, 20-26	20, 20-28	20, 20-28
Quartiles (25, 50, 75)	20, 20, 21	20, 20, 21	20, 20, 21
T3 (30 weeks)	n=36	n=29	n=65
Mean (SD)	32.1 (2.8)	32.0 (3.1)	32.1 (2.9)
Median, range	31, 30-44	31, 30-43	31, 30-44
Quartiles (25, 50, 75)	30, 31, 33	30, 31, 32	30, 31, 33

[a]CBT: Cognitive behavioral therapy; SD: standard deviation.
