# Supplementary material for: Cost and Effectiveness of Blended Versus Standard Cognitive Behavioral Therapy for Outpatients With Depression in Routine Specialized Mental Health Care: Pilot Randomized Controlled Trial
Source: J Med Internet Res. 2019 Oct 29;21(10):e14261. doi: 10.2196/14261 (PMC6914243; doi:10.2196/14261)
Supplement: Multimedia Appendix 3 [file jmir_v21i10e14261_app3.pdf]

CONSORT-EHEALTH (V 1.6.1) - Submission/Publication Form

The CONSORT-EHEALTH checklist is intended for authors of randomized trials evaluating web-based and Internet-based applications/interventions, including mobile interventions, electronic games (incl multiplayer games), social media, certain telehealth applications, and other interactive and/or networked electronic applications. Some of the items (e.g. all subitems under item 5 - description of the intervention) may also be applicable for other study designs.

The goal of the CONSORT EHEALTH checklist and guideline is to be
a) a guide for reporting for authors of RCTs,
b) to form a basis for appraisal of an ehealth trial (in terms of validity)

CONSORT-EHEALTH items/subitems are MANDATORY reporting items for studies published in the Journal of Medical Internet Research and other journals / scientific societies endorsing the checklist.

Items numbered 1., 2., 3., 4a., 4b etc are original CONSORT or CONSORT-NPT (non-pharmacologic treatment) items.
Items with Roman numerals (i., ii, iii, iv etc.) are CONSORT-EHEALTH extensions/clarifications.

As the CONSORT-EHEALTH checklist is still considered in a formative stage, we would ask that you also RATE ON A SCALE OF 1-5 how important/useful you feel each item is FOR THE PURPOSE OF THE CHECKLIST and reporting guideline (optional).

Mandatory reporting items are marked with a red *.
In the textboxes, either copy & paste the relevant sections from your manuscript into this form - please include any quotes from your manuscript in QUOTATION MARKS,
or answer directly by providing additional information not in the manuscript, or elaborating on why the item was not relevant for this study.

YOUR ANSWERS WILL BE PUBLISHED AS A SUPPLEMENTARY FILE TO YOUR PUBLICATION IN JMIR AND ARE CONSIDERED PART OF YOUR PUBLICATION (IF ACCEPTED).
Please fill in these questions diligently. Information will not be copyedited, so please use proper spelling and grammar, use correct capitalization, and avoid abbreviations.

DO NOT FORGET TO SAVE AS PDF _AND_ CLICK THE SUBMIT BUTTON SO YOUR ANSWERS ARE IN OUR DATABASE !!!

Citation Suggestion (if you append the pdf as Appendix we suggest to cite this paper in the caption):
Eysenbach G, CONSORT-EHEALTH Group
CONSORT-EHEALTH: Improving and Standardizing Evaluation Reports of Web-based and Mobile Health Interventions
J Med Internet Res 2011;13(4):e126
URL: http://www.jmir.org/2011/4/e126/
doi: 10.2196/jmir.1923
PMID: 22209829

* Required

Your name *

First Last

Lisa Kooistra

Primary Affiliation (short), City, Country *

University of Toronto, Toronto, Canada

GGZ inGeest/Vumc, Amsterdam, The Ne

Your e-mail address *

abc@gmail.com

l.kooistra@ggzingeest.nl

Title of your manuscript *

Provide the (draft) title of your manuscript.

Costs and effectiveness of blended versus standard cognitive behavioural therapy for depressed outpatients in routine specialised mental healthcare: a pilot randomised controlled trial

Name of your App/Software/Intervention *

If there is a short and a long/alternate name, write the short name first and add the long name in brackets.

bCBT, blended cognitive behavioural ther

Evaluated Version (if any)

e.g. "V1", "Release 2017-03-01", "Version 2.0.27913"

Your answer

Language(s) *

What language is the intervention/app in? If multiple languages are available, separate by comma (e.g. "English, French")

Dutch

URL of your Intervention Website or App

e.g. a direct link to the mobile app on app in appstore (itunes, Google Play), or URL of the website. If the intervention is a DVD or hardware, you can also link to an Amazon page.

Your answer

URL of an image/screenshot (optional)

Your answer

Accessibility *

Can an enduser access the intervention presently?

- ○ access is free and open
- ◉ access only for special usergroups, not open
- ○ access is open to everyone, but requires payment/subscription/in-app purchases
- ○ app/intervention no longer accessible
- ○ Other:

Primary Medical Indication/Disease/Condition *

e.g. "Stress", "Diabetes", or define the target group in brackets after the condition, e.g. "Autism (Parents of children with)", "Alzheimers (Informal Caregivers of)"

Depression

Primary Outcomes measured in trial *

comma-separated list of primary outcomes reported in the trial

Self-reported depression severity (IDS-SF

Secondary/other outcomes

Are there any other outcomes the intervention is expected to affect?

Your answer

Recommended "Dose" *

What do the instructions for users say on how often the app should be used?

- ○ Approximately Daily
- ◉ Approximately Weekly
- ○ Approximately Monthly
- ○ Approximately Yearly
- ○ "as needed"
- ○ Other:

Approx. Percentage of Users (starters) still using the app as recommended after 3 months *

- (selected) unknown / not evaluated
- 0-10%
- 11-20%
- 21-30%
- 31-40%
- 41-50%
- 51-60%
- 61-70%
- 71%-80%
- 81-90%
- 91-100%
- Other:

Overall, was the app/intervention effective? *

- ○ yes: all primary outcomes were significantly better in intervention group vs control
- ○ partly: SOME primary outcomes were significantly better in intervention group vs control
- ◉ no statistically significant difference between control and intervention
- ○ potentially harmful: control was significantly better than intervention in one or more outcomes
- ○ inconclusive: more research is needed
- ○ Other:

Article Preparation Status/Stage *

At which stage in your article preparation are you currently (at the time you fill in this form)

- ○ not submitted yet - in early draft status
- ◉ not submitted yet - in late draft status, just before submission
- ○ submitted to a journal but not reviewed yet
- ○ submitted to a journal and after receiving initial reviewer comments
- ○ submitted to a journal and accepted, but not published yet
- ○ published
- ○ Other:

Journal *

If you already know where you will submit this paper (or if it is already submitted), please provide the journal name (if it is not JMIR, provide the journal name under "other")

- ( ) not submitted yet / unclear where I will submit this
- (x) Journal of Medical Internet Research (JMIR)
- ( ) JMIR mHealth and UHealth
- ( ) JMIR Serious Games
- ( ) JMIR Mental Health
- ( ) JMIR Public Health
- ( ) JMIR Formative Research
- ( ) Other JMIR sister journal
- ( ) Other:

Is this a full powered effectiveness trial or a pilot/feasibility trial? *

- (x) Pilot/feasibility
- ( ) Fully powered

Manuscript tracking number *

If this is a JMIR submission, please provide the manuscript tracking number under "other" (The ms tracking number can be found in the submission acknowledgement email, or when you login as author in JMIR. If the paper is already published in JMIR, then the ms tracking number is the four-digit number at the end of the DOI, to be found at the bottom of each published article in JMIR)

- (x) no ms number (yet) / not (yet) submitted to / published in JMIR
- ( ) Other:

TITLE AND ABSTRACT

1a) TITLE: Identification as a randomized trial in the title

1a) Does your paper address CONSORT item 1a? *

I.e does the title contain the phrase "Randomized Controlled Trial"? (if not, explain the reason under "other")

- (selected) yes
- Other:

1a-i) Identify the mode of delivery in the title

Identify the mode of delivery. Preferably use “web-based” and/or “mobile” and/or “electronic game” in the title. Avoid ambiguous terms like “online”, “virtual”, “interactive”. Use “Internet-based” only if Intervention includes non-web-based Internet components (e.g. email), use “computer-based” or “electronic” only if offline products are used. Use “virtual” only in the context of “virtual reality” (3-D worlds). Use “online” only in the context of “online support groups”. Complement or substitute product names with broader terms for the class of products (such as “mobile” or “smart phone” instead of “iphone”), especially if the application runs on different platforms.

	1	2	3	4	5	
subitem not at all important	○	●	○	○	○	essential

Does your paper address subitem 1a-i? *

Copy and paste relevant sections from manuscript title (include quotes in quotation marks "like this" to indicate direct quotes from your manuscript), or elaborate on this item by providing additional information not in the ms, or briefly explain why the item is not applicable/relevant for your study

The term "blended" was added to the title

1a-ii) Non-web-based components or important co-interventions in title

Mention non-web-based components or important co-interventions in title, if any (e.g., “with telephone support”).

	1	2	3	4	5	
subitem not at all important	○	●	○	○	○	essential

Does your paper address subitem 1a-ii?

Copy and paste relevant sections from manuscript title (include quotes in quotation marks "like this" to indicate direct quotes from your manuscript), or elaborate on this item by providing additional information not in the ms, or briefly explain why the item is not applicable/relevant for your study

Your answer

1a-iii) Primary condition or target group in the title

Mention primary condition or target group in the title, if any (e.g., “for children with Type I Diabetes”) Example: A Web-based and Mobile Intervention with Telephone Support for Children with Type I Diabetes: Randomized Controlled Trial

	1	2	3	4	5	
subitem not at all important	○	○	○	◉	○	essential

Does your paper address subitem 1a-iii? *

Copy and paste relevant sections from manuscript title (include quotes in quotation marks "like this" to indicate direct quotes from your manuscript), or elaborate on this item by providing additional information not in the ms, or briefly explain why the item is not applicable/relevant for your study

"depressed outpatients in routine specialised mental healthcare"

1b) ABSTRACT: Structured summary of trial design, methods, results, and conclusions

NPT extension: Description of experimental treatment, comparator, care providers, centers, and blinding status.

1b-i) Key features/functionalities/components of the intervention and comparator in the METHODS section of the ABSTRACT

Mention key features/functionalities/components of the intervention and comparator in the abstract. If possible, also mention theories and principles used for designing the site. Keep in mind the needs of systematic reviewers and indexers by including important synonyms. (Note: Only report in the abstract what the main paper is reporting. If this information is missing from the main body of text, consider adding it)

	1	2	3	4	5	
subitem not at all important	○	○	○	◉	○	essential

Does your paper address subitem 1b-i? *

Copy and paste relevant sections from the manuscript abstract (include quotes in quotation marks "like this" to indicate direct quotes from your manuscript), or elaborate on this item by providing additional information not in the ms, or briefly explain why the item is not applicable/relevant for your study

"Blended CBT consisted of 10 weekly face-to-face sessions at a specialised mental healthcare clinic and 9 web-based sessions that patients worked through at home. Standard CBT consisted of 15 to 20 weekly face-to-face sessions."

1b-ii) Level of human involvement in the METHODS section of the ABSTRACT

Clarify the level of human involvement in the abstract, e.g., use phrases like "fully automated" vs. "therapist/nurse/care provider/physician-assisted" (mention number and expertise of providers involved, if any). (Note: Only report in the abstract what the main paper is reporting. If this information is missing from the main body of text, consider adding it)

	1	2	3	4	5	
subitem not at all important	○	○	○	◉	○	essential

Does your paper address subitem 1b-ii?

Copy and paste relevant sections from the manuscript abstract (include quotes in quotation marks "like this" to indicate direct quotes from your manuscript), or elaborate on this item by providing additional information not in the ms, or briefly explain why the item is not applicable/relevant for your study

Yes, the term blended is used

1b-iii) Open vs. closed, web-based (self-assessment) vs. face-to-face assessments in the METHODS section of the ABSTRACT

Mention how participants were recruited (online vs. offline), e.g., from an open access website or from a clinic or a closed online user group (closed usergroup trial), and clarify if this was a purely web-based trial, or there were face-to-face components (as part of the intervention or for assessment). Clearly say if outcomes were self-assessed through questionnaires (as common in web-based trials). Note: In traditional offline trials, an open trial (open-label trial) is a type of clinical trial in which both the researchers and participants know which treatment is being administered. To avoid confusion, use "blinded" or "unblinded" to indicated the level of blinding instead of "open", as "open" in web-based trials usually refers to "open access" (i.e. participants can self-enrol). (Note: Only report in the abstract what the main paper is reporting. If this information is missing from the main body of text, consider adding it)

	1	2	3	4	5	
subitem not at all important	○	○	○	○	○	essential

Does your paper address subitem 1b-iii?

Copy and paste relevant sections from the manuscript abstract (include quotes in quotation marks "like this" to indicate direct quotes from your manuscript), or elaborate on this item by providing additional information not in the ms, or briefly explain why the item is not applicable/relevant for your study

"At baseline, and 10, 20 and 30 weeks after start of treatment, self-assessed depression severity (IDS-SR), quality-adjusted life-years (EQ-5D-3L) and costs (TiC-P) were measured. In addition, clinicians conducted diagnostic interviews (M.I.N.I. plus) at all time-points, evaluating presence of depressive episodes. At baseline and 30-week follow-up, depression was assessed along with other co-morbid psychiatric disorders. Assessors were blinded to treatment allocation. "

1b-iv) RESULTS section in abstract must contain use data

Report number of participants enrolled/assessed in each group, the use/uptake of the intervention (e.g., attrition/adherence metrics, use over time, number of logins etc.), in addition to primary/secondary outcomes. (Note: Only report in the abstract what the main paper is reporting. If this information is missing from the main body of text, consider adding it)

	1	2	3	4	5	
subitem not at all important	○	○	○	●	○	essential

Does your paper address subitem 1b-iv?

Copy and paste relevant sections from the manuscript abstract (include quotes in quotation marks "like this" to indicate direct quotes from your manuscript), or elaborate on this item by providing additional information not in the ms, or briefly explain why the item is not applicable/relevant for your study

"Depressed patients in specialised mental healthcare were randomly allocated to blended CBT (bCBT; n=53) or standard CBT (n=49)".

"Patients in bCBT had high treatment adherence rates (85%). Treatment duration in bCBT was 19.0 weeks [SD 12.6] versus 33.2 weeks [SD 23.0] in standard CBT (p<.001)".

1b-v) CONCLUSIONS/DISCUSSION in abstract for negative trials

Conclusions/Discussions in abstract for negative trials: Discuss the primary outcome - if the trial is negative (primary outcome not changed), and the intervention was not used, discuss whether negative results are attributable to lack of uptake and discuss reasons. (Note: Only report in the abstract what the main paper is reporting. If this information is missing from the main body of text, consider adding it)

	1	2	3	4	5	
subitem not at all important	○	○	○	○	◉	essential

Does your paper address subitem 1b-v?

Copy and paste relevant sections from the manuscript abstract (include quotes in quotation marks "like this" to indicate direct quotes from your manuscript), or elaborate on this item by providing additional information not in the ms, or briefly explain why the item is not applicable/relevant for your study

" Results: From a societal perspective, bCBT was not cost-effective compared to standard CBT (1,183, 95% CI 399 to 2,765). From a healthcare perspective, bCBT had a high probability (.75) of being cost-saving (-176, 95% CI -659 to 343). BCBT had a high probability of generating treatment response and better quality of life, but a low probability of avoiding depressive episodes compared to standard CBT. Patients in bCBT had high treatment adherence rates (85%). Treatment duration in bCBT was 19.0 weeks [SD 12.6] versus 33.2 weeks [SD 23.0] in standard CBT (p<.001). Groups were comparable across all outcomes.
Conclusions: This pilot study shows that bCBT might be a promising way to engage depressed patients in specialised mental healthcare. Results should be carefully interpreted, due to the small sample size. Further research in larger replication studies is warranted - focussing on optimising the clinical effects of bCBT, as well as its budget impact. "

INTRODUCTION

2a) In INTRODUCTION: Scientific background and explanation of rationale

2a-i) Problem and the type of system/solution

Describe the problem and the type of system/solution that is object of the study: intended as stand-alone intervention vs. incorporated in broader health care program? Intended for a particular patient population? Goals of the intervention, e.g., being more cost-effective to other interventions, replace or complement other solutions? (Note: Details about the intervention are provided in “Methods” under 5)

	1	2	3	4	5	
subitem not at all important	○	○	○	○	◉	essential

Does your paper address subitem 2a-i? *

Copy and paste relevant sections from the manuscript (include quotes in quotation marks "like this" to indicate direct quotes from your manuscript), or elaborate on this item by providing additional information not in the ms, or briefly explain why the item is not applicable/relevant for your study

"The current study compared costs and effects of integrated blended CBT (bCBT) to standard CBT, when provided to depressed patients in the acute phase of treatment in specialised mental healthcare. The blended treatment aimed to replace half of face-to-face treatment with online sessions, and thereby shorten treatment duration when compared to standard CBT for Major Depressive Disorder [21]. The study examined whether bCBT can lead to comparable clinical effects as standard CBT, at lower costs. The study was designed as a pilot study with the aim to guide further development of blended CBT and inform future research on feasibility, effectiveness and cost-effectiveness of bCBT for depression in outpatient specialised mental healthcare."

2a-ii) Scientific background, rationale: What is known about the (type of) system

Scientific background, rationale: What is known about the (type of) system that is the object of the study (be sure to discuss the use of similar systems for other conditions/diagnoses, if appropiate), motivation for the study, i.e. what are the reasons for and what is the context for this specific study, from which stakeholder viewpoint is the study performed, potential impact of findings [2]. Briefly justify the choice of the comparator.

	1	2	3	4	5	
subitem not at all important	○	○	○	○	◉	essential

Does your paper address subitem 2a-ii? *

Copy and paste relevant sections from the manuscript (include quotes in quotation marks "like this" to indicate direct quotes from your manuscript), or elaborate on this item by providing additional information not in the ms, or briefly explain why the item is not applicable/relevant for your study

"Web-based (online) psychotherapy is often cited as a promising way to reduce treatment costs and increase treatment availability for common mental disorders [7–10], for example by lowering the required therapist time per patient [11]. The efficacy of several formats has been validated for the treatment of depression when compared to control groups [12–14]. Evidence suggests that therapist-guided online treatment can be equally as effective as standard face-to-face therapy [15,16]. The cost-effectiveness of guided online treatment has been less well studied. An individual patient data meta-analysis (IPD-MA), pooling the available estimates from five RCTs, suggested that guided online treatment is not (yet) cost-effective compared to control conditions [17].

To date, most studies focused on community samples and self-referred participants. Less is known about the costs and effects of online therapy in routine specialised mental healthcare for patients with more severe or complex depression profiles. This patient group is likely to require more personalised treatment, for example by monitoring suicidal ideation or addressing co-morbid disorders [15]. The integration of online and face-to-face treatment into a blended treatment format could be a promising way to address these matters [18–22]. Blended treatment allows therapists to closely monitor their patients, both in face-to-face sessions at the clinic and in an online environment. At the same time, it is thought to retain positive aspects associated with online treatment, such as lower costs compared to standard treatment, reduction of therapist time and increased patient self-management [18,23].

Current evidence for blended therapy is limited, although several studies are in progress [24–27]. Most studies to date evaluated the online component as an add-on to standard care, rather than offering an integrated blended treatment protocol [28–32]. Results of these studies are promising, and indicate that blended treatment may be a viable treatment option. However, the treatment format limits the margin for cost-effectiveness, as therapist time is not reduced and overall treatment-dosage may even increase [17,33]."

2b) In INTRODUCTION: Specific objectives or hypotheses

Does your paper address CONSORT subitem 2b? *

Copy and paste relevant sections from the manuscript (include quotes in quotation marks "like this" to indicate direct quotes from your manuscript), or elaborate on this item by providing additional information not in the ms, or briefly explain why the item is not applicable/relevant for your study

"The current study compared costs and effects of integrated blended CBT (bCBT) to standard CBT, when provided to depressed patients in the acute phase of treatment in specialised mental healthcare. The blended treatment aimed to replace half of face-to-face treatment with online sessions, and thereby shorten treatment duration when compared to standard CBT for Major Depressive Disorder [21]. The study examined whether bCBT can lead to comparable clinical effects as standard CBT, at lower costs. The study was designed as a pilot study with the aim to guide further development of blended CBT and inform future research on feasibility, effectiveness and cost-effectiveness of bCBT for depression in outpatient specialised mental healthcare."

METHODS

3a) Description of trial design (such as parallel, factorial) including allocation ratio

Does your paper address CONSORT subitem 3a? *

Copy and paste relevant sections from the manuscript (include quotes in quotation marks "like this" to indicate direct quotes from your manuscript), or elaborate on this item by providing additional information not in the ms, or briefly explain why the item is not applicable/relevant for your study

"This pilot study was designed as a randomised controlled trial in three outpatient specialised mental healthcare clinics in the Netherlands, at five different locations. Blended CBT was compared to standard CBT for depression."

"Patients were randomly allocated to bCBT or standard CBT when they met inclusion criteria, provided a signed informed consent form and completed the full baseline assessment. Allocation was stratified by mental healthcare centre using a computer-generated random number table. An independent researcher conducted the randomisation. Patients and therapists were aware of group allocation, but allocation was concealed from outcome assessors. "

3b) Important changes to methods after trial commencement (such as eligibility criteria), with reasons

Does your paper address CONSORT subitem 3b? *

Copy and paste relevant sections from the manuscript (include quotes in quotation marks "like this" to indicate direct quotes from your manuscript), or elaborate on this item by providing additional information not in the ms, or briefly explain why the item is not applicable/relevant for your study

-

3b-i) Bug fixes, Downtimes, Content Changes

Bug fixes, Downtimes, Content Changes: ehealth systems are often dynamic systems. A description of changes to methods therefore also includes important changes made on the intervention or comparator during the trial (e.g., major bug fixes or changes in the functionality or content) (5-iii) and other “unexpected events” that may have influenced study design such as staff changes, system failures/downtimes, etc. [2].

	1	2	3	4	5	
subitem not at all important	○	○	◉	○	○	essential

Does your paper address subitem 3b-i?

Copy and paste relevant sections from the manuscript (include quotes in quotation marks "like this" to indicate direct quotes from your manuscript), or elaborate on this item by providing additional information not in the ms, or briefly explain why the item is not applicable/relevant for your study

No

4a) Eligibility criteria for participants

Does your paper address CONSORT subitem 4a? *

Copy and paste relevant sections from the manuscript (include quotes in quotation marks "like this" to indicate direct quotes from your manuscript), or elaborate on this item by providing additional information not in the ms, or briefly explain why the item is not applicable/relevant for your study

"Adult patients with a DSM-IV-TR diagnosis [34] of Major Depressive Disorder (MDD), who were indicated for individual CBT by the local intake staff, were recruited during the intake procedure. In the Netherlands, patients can be referred to specialised services when they do not respond to treatment in primary mental healthcare [35]. Therefore, patients in this trial were likely to have complex or severe clinical profiles, and to have undergone some form of pharmacotherapy or short-term psychotherapy prior to the current study.
Exclusion criteria were inadequate proficiency in the Dutch language, no valid e-mail address or no computer with Internet access, current psychotic disorder, bipolar disorder or substance dependence, or a high risk for suicide (current plans). Patients with current substance dependence could participate in the trial if they reported abstinence and had been treated at a centre specialised in treatment of substance abuse for at least a month prior to random allocation."

4a-i) Computer / Internet literacy

Computer / Internet literacy is often an implicit “de facto” eligibility criterion - this should be explicitly clarified.

	1	2	3	4	5	
subitem not at all important	○	○	●	○	○	essential

Does your paper address subitem 4a-i?

Copy and paste relevant sections from the manuscript (include quotes in quotation marks "like this" to indicate direct quotes from your manuscript), or elaborate on this item by providing additional information not in the ms, or briefly explain why the item is not applicable/relevant for your study

No computer literacy was required, but people were excluded if they did not have a valid e-mail address or computer with Internet access

4a-ii) Open vs. closed, web-based vs. face-to-face assessments:

Open vs. closed, web-based vs. face-to-face assessments: Mention how participants were recruited (online vs. offline), e.g., from an open access website or from a clinic, and clarify if this was a purely web-based trial, or there were face-to-face components (as part of the intervention or for assessment), i.e., to what degree got the study team to know the participant. In online-only trials, clarify if participants were quasi-anonymous and whether having multiple identities was possible or whether technical or logistical measures (e.g., cookies, email confirmation, phone calls) were used to detect/prevent these.

	1	2	3	4	5	
subitem not at all important	○	○	○	○	○	essential

Does your paper address subitem 4a-ii? *

Copy and paste relevant sections from the manuscript (include quotes in quotation marks "like this" to indicate direct quotes from your manuscript), or elaborate on this item by providing additional information not in the ms, or briefly explain why the item is not applicable/relevant for your study

Offline: "Adult patients with a DSM-IV-TR diagnosis [34] of Major Depressive Disorder (MDD), who were indicated for individual CBT by the local intake staff, were recruited during the intake procedure"

4a-iii) Information giving during recruitment

Information given during recruitment. Specify how participants were briefed for recruitment and in the informed consent procedures (e.g., publish the informed consent documentation as appendix, see also item X26), as this information may have an effect on user self-selection, user expectation and may also bias results.

	1	2	3	4	5	
subitem not at all important	○	○	○	○	○	essential

Does your paper address subitem 4a-iii?

Copy and paste relevant sections from the manuscript (include quotes in quotation marks "like this" to indicate direct quotes from your manuscript), or elaborate on this item by providing additional information not in the ms, or briefly explain why the item is not applicable/relevant for your study

"Patients were randomly allocated to bCBT or standard CBT when they met inclusion criteria, provided a signed informed consent form and completed the full baseline assessment. "

4b) Settings and locations where the data were collected

Does your paper address CONSORT subitem 4b? *

Copy and paste relevant sections from the manuscript (include quotes in quotation marks "like this" to indicate direct quotes from your manuscript), or elaborate on this item by providing additional information not in the ms, or briefly explain why the item is not applicable/relevant for your study

"This pilot study was designed as a randomised controlled trial in three outpatient specialised mental healthcare clinics in the Netherlands, at five different locations. "

4b-i) Report if outcomes were (self-)assessed through online questionnaires

Clearly report if outcomes were (self-)assessed through online questionnaires (as common in web-based trials) or otherwise.

	1	2	3	4	5	
subitem not at all important	○	○	○	○	○	essential

Does your paper address subitem 4b-i? *

Copy and paste relevant sections from the manuscript (include quotes in quotation marks "like this" to indicate direct quotes from your manuscript), or elaborate on this item by providing additional information not in the ms, or briefly explain why the item is not applicable/relevant for your study

"The primary clinical outcome was self-reported depression severity", "as measured by a web-based version of the Inventory of Depressive Symptomatology"
"Clinician-rated outcomes included presence of a depressive episode at 10, 20 and 30 weeks after start of treatment and during follow-up. Depression diagnosis was assessed at 10, 20 and 30 weeks after the start of treatment during a telephone interview with section A of the M.I.N.I. plus diagnostic interview [36,37] by trained research assistants who were blinded to treatment allocation. At baseline and 30-week follow-up, all sections of the M.I.N.I plus interview were administered during a face-to-face or telephone interview, to assess co-morbid diagnoses. "
"In order to calculate quality-adjusted life-years (QALYs), the EuroQol (EQ-5D-3L) [48] questionnaire was administered online at all four time-points."
" Self-reported resource use in the four weeks prior to each assessment was measured with a web-based version of the Trimbos and iMTA questionnaire on Costs associated with Psychiatric illness (TiC-P) questionnaire [50]."

4b-ii) Report how institutional affiliations are displayed

Report how institutional affiliations are displayed to potential participants [on ehealth media], as affiliations with prestigious hospitals or universities may affect volunteer rates, use, and reactions with regards to an intervention.(Not a required item – describe only if this may bias results)

	1	2	3	4	5	
subitem not at all important	○	○	○	○	○	essential

Does your paper address subitem 4b-ii?

Copy and paste relevant sections from the manuscript (include quotes in quotation marks "like this" to indicate direct quotes from your manuscript), or elaborate on this item by providing additional information not in the ms, or briefly explain why the item is not applicable/relevant for your study

Your answer

5) The interventions for each group with sufficient details to allow replication, including how and when they were actually administered

5-i) Mention names, credential, affiliations of the developers, sponsors, and owners

Mention names, credential, affiliations of the developers, sponsors, and owners [6] (if authors/evaluators are owners or developer of the software, this needs to be declared in a “Conflict of interest” section or mentioned elsewhere in the manuscript).

	1	2	3	4	5	
subitem not at all important	○	○	○	○	○	essential

Does your paper address subitem 5-i?

Copy and paste relevant sections from the manuscript (include quotes in quotation marks "like this" to indicate direct quotes from your manuscript), or elaborate on this item by providing additional information not in the ms, or briefly explain why the item is not applicable/relevant for your study

Your answer

5-ii) Describe the history/development process

Describe the history/development process of the application and previous formative evaluations (e.g., focus groups, usability testing), as these will have an impact on adoption/use rates and help with interpreting results.

	1	2	3	4	5	
subitem not at all important	○	○	○	○	○	essential

Does your paper address subitem 5-ii?

Copy and paste relevant sections from the manuscript (include quotes in quotation marks "like this" to indicate direct quotes from your manuscript), or elaborate on this item by providing additional information not in the ms, or briefly explain why the item is not applicable/relevant for your study

We refer to a specific paper that was written about the development process.

5-iii) Revisions and updating

Revisions and updating. Clearly mention the date and/or version number of the application/intervention (and comparator, if applicable) evaluated, or describe whether the intervention underwent major changes during the evaluation process, or whether the development and/or content was "frozen" during the trial. Describe dynamic components such as news feeds or changing content which may have an impact on the replicability of the intervention (for unexpected events see item 3b).

	1	2	3	4	5	
subitem not at all important	○	○	○	○	○	essential

Does your paper address subitem 5-iii?

Copy and paste relevant sections from the manuscript (include quotes in quotation marks "like this" to indicate direct quotes from your manuscript), or elaborate on this item by providing additional information not in the ms, or briefly explain why the item is not applicable/relevant for your study

Not described in the paper

5-iv) Quality assurance methods

Provide information on quality assurance methods to ensure accuracy and quality of information provided [1], if applicable.

	1	2	3	4	5	
subitem not at all important	○	○	○	○	○	essential

Does your paper address subitem 5-iv?

Copy and paste relevant sections from the manuscript (include quotes in quotation marks "like this" to indicate direct quotes from your manuscript), or elaborate on this item by providing additional information not in the ms, or briefly explain why the item is not applicable/relevant for your study

Not applicable

5-v) Ensure replicability by publishing the source code, and/or providing screenshots/screen-capture video, and/or providing flowcharts of the algorithms used

Ensure replicability by publishing the source code, and/or providing screenshots/screen-capture video, and/or providing flowcharts of the algorithms used. Replicability (i.e., other researchers should in principle be able to replicate the study) is a hallmark of scientific reporting.

	1	2	3	4	5	
subitem not at all important	○	○	○	○	○	essential

Does your paper address subitem 5-v?

Copy and paste relevant sections from the manuscript (include quotes in quotation marks "like this" to indicate direct quotes from your manuscript), or elaborate on this item by providing additional information not in the ms, or briefly explain why the item is not applicable/relevant for your study

The content is not open-source.

5-vi) Digital preservation

Digital preservation: Provide the URL of the application, but as the intervention is likely to change or disappear over the course of the years; also make sure the intervention is archived (Internet Archive, webcitation.org, and/or publishing the source code or screenshots/videos alongside the article). As pages behind login screens cannot be archived, consider creating demo pages which are accessible without login.

	1	2	3	4	5	
subitem not at all important	○	○	○	○	○	essential

Does your paper address subitem 5-vi?

Copy and paste relevant sections from the manuscript (include quotes in quotation marks "like this" to indicate direct quotes from your manuscript), or elaborate on this item by providing additional information not in the ms, or briefly explain why the item is not applicable/relevant for your study

No

5-vii) Access

Access: Describe how participants accessed the application, in what setting/context, if they had to pay (or were paid) or not, whether they had to be a member of specific group. If known, describe how participants obtained “access to the platform and Internet” [1]. To ensure access for editors/reviewers/readers, consider to provide a “backdoor” login account or demo mode for reviewers/readers to explore the application (also important for archiving purposes, see vi).

	1	2	3	4	5	
subitem not at all important	○	○	○	○	○	essential

Does your paper address subitem 5-vii? *

Copy and paste relevant sections from the manuscript (include quotes in quotation marks "like this" to indicate direct quotes from your manuscript), or elaborate on this item by providing additional information not in the ms, or briefly explain why the item is not applicable/relevant for your study

"Therapists and patients had personal, password-protected, accounts on the web-based treatment platform. Therapists were trained in the use of the web-based treatment platform and received a treatment manual. Patients received information on how to work with the platform in the first face-to-face session. "

5-viii) Mode of delivery, features/functionalities/components of the intervention and comparator, and the theoretical framework

Describe mode of delivery, features/functionalities/components of the intervention and comparator, and the theoretical framework [6] used to design them (instructional strategy [1], behaviour change techniques, persuasive features, etc., see e.g., [7, 8] for terminology). This includes an in-depth description of the content (including where it is coming from and who developed it) [1],” whether [and how] it is tailored to individual circumstances and allows users to track their progress and receive feedback” [6]. This also includes a description of communication delivery channels and – if computer-mediated communication is a component – whether communication was synchronous or asynchronous [6]. It also includes information on presentation strategies [1], including page design principles, average amount of text on pages, presence of hyperlinks to other resources, etc. [1].

	1	2	3	4	5	
subitem not at all important	○	○	○	○	○	essential

Does your paper address subitem 5-viii? *

Copy and paste relevant sections from the manuscript (include quotes in quotation marks "like this" to indicate direct quotes from your manuscript), or elaborate on this item by providing additional information not in the ms, or briefly explain why the item is not applicable/relevant for your study

"Both interventions consisted of cognitive behavioural depression treatment. The content of CBT was based on the standard treatment manual [39–41], which recommends 15 to 20 weekly sessions focusing on psycho-education, behavioural activation, cognitive restructuring and relapse prevention. Patients who completed 14 sessions or more (75% of 18 sessions) were considered treatment completers. Parallel pharmacological treatment was allowed.
Blended CBT consisted of 10 face-to-face sessions at the specialised mental healthcare centre and 9 web-based (online) sessions that patients worked through at home. Therapists and patients had personal, password-protected, accounts on the web-based treatment platform. Therapists were trained in the use of the web-based treatment platform and received a treatment manual. Patients received information on how to work with the platform in the first face-to-face session. After each online session, therapists provided therapeutic feedback regarding content and progress. Therapists could let patients repeat an online session if this was warranted. The bCBT protocol aimed to provide one face-to-face, one online session and one online feedback message per week during a 10-week period, starting with a face-to-face session. The intervention was semi-structured, offering online sessions in a fixed order and providing therapists with a protocol for each face-to-face session. Therapists were allowed to personalise face-to-face sessions to each patient's individual needs and situation in terms of themes and techniques. More detailed information on the format and content of bCBT can be found elsewhere [21,38].
The standard CBT group received individual face-to-face cognitive behavioural depression treatment at the clinic, in accordance with routine care procedures. The content of standard CBT was comparable to the content of bCBT. Therapists were advised to plan weekly sessions, but were allowed to deviate from the treatment manual when necessary. Based on the guideline, standard CBT was expected to consist of 18 sessions on average, provided over a period of approximately 20 weeks. However, duration of treatment could vary per patient."

5-ix) Describe use parameters

Describe use parameters (e.g., intended "doses" and optimal timing for use). Clarify what instructions or recommendations were given to the user, e.g., regarding timing, frequency, heaviness of use, if any, or was the intervention used ad libitum.

	1	2	3	4	5	
subitem not at all important	○	○	○	○	○	essential

Does your paper address subitem 5-ix?

Copy and paste relevant sections from the manuscript (include quotes in quotation marks "like this" to indicate direct quotes from your manuscript), or elaborate on this item by providing additional information not in the ms, or briefly explain why the item is not applicable/relevant for your study

Yes, see 5-viii

5-x) Clarify the level of human involvement

Clarify the level of human involvement (care providers or health professionals, also technical assistance) in the e-intervention or as co-intervention (detail number and expertise of professionals involved, if any, as well as “type of assistance offered, the timing and frequency of the support, how it is initiated, and the medium by which the assistance is delivered”. It may be necessary to distinguish between the level of human involvement required for the trial, and the level of human involvement required for a routine application outside of a RCT setting (discuss under item 21 – generalizability).

	1	2	3	4	5	
subitem not at all important	○	○	○	○	○	essential

Does your paper address subitem 5-x?

Copy and paste relevant sections from the manuscript (include quotes in quotation marks "like this" to indicate direct quotes from your manuscript), or elaborate on this item by providing additional information not in the ms, or briefly explain why the item is not applicable/relevant for your study

Yes, see 5-viii

5-xi) Report any prompts/reminders used

Report any prompts/reminders used: Clarify if there were prompts (letters, emails, phone calls, SMS) to use the application, what triggered them, frequency etc. It may be necessary to distinguish between the level of prompts/reminders required for the trial, and the level of prompts/reminders for a routine application outside of a RCT setting (discuss under item 21 – generalizability).

	1	2	3	4	5	
subitem not at all important	○	○	○	○	○	essential

Does your paper address subitem 5-xi? *

Copy and paste relevant sections from the manuscript (include quotes in quotation marks "like this" to indicate direct quotes from your manuscript), or elaborate on this item by providing additional information not in the ms, or briefly explain why the item is not applicable/relevant for your study

Not addressed in the paper. Participants received automated e-mail reminders from the web-based platform, informing them when a new online session, new therapist feedback or a new message was available. Participants could also choose to turn this option off.

5-xii) Describe any co-interventions (incl. training/support)

Describe any co-interventions (incl. training/support): Clearly state any interventions that are provided in addition to the targeted eHealth intervention, as ehealth intervention may not be designed as stand-alone intervention. This includes training sessions and support [1]. It may be necessary to distinguish between the level of training required for the trial, and the level of training for a routine application outside of a RCT setting (discuss under item 21 – generalizability.

	1	2	3	4	5	
subitem not at all important	○	○	○	○	○	essential

Does your paper address subitem 5-xii? *

Copy and paste relevant sections from the manuscript (include quotes in quotation marks "like this" to indicate direct quotes from your manuscript), or elaborate on this item by providing additional information not in the ms, or briefly explain why the item is not applicable/relevant for your study

Yes, see 5-viii

6a) Completely defined pre-specified primary and secondary outcome measures, including how and when they were assessed

Does your paper address CONSORT subitem 6a? *

Copy and paste relevant sections from the manuscript (include quotes in quotation marks "like this" to indicate direct quotes from your manuscript), or elaborate on this item by providing additional information not in the ms, or briefly explain why the item is not applicable/relevant for your study

he primary clinical outcome was self-reported depression severity", "as measured by a web-based version of the Inventory of Depressive Symptomatology"
"Within the cost-effectiveness framework, the IDS-SR30 scores were used to assess treatment response, based on the reliable change (RC) index [46]."
"Clinician-rated outcomes included presence of a depressive episode at 10, 20 and 30 weeks after start of treatment and during follow-up. Depression diagnosis was assessed at 10, 20 and 30 weeks after the start of treatment during a telephone interview with section A of the M.I.N.I. plus diagnostic interview [36,37] by trained research assistants who were blinded to treatment allocation. At baseline and 30-week follow-up, all sections of the M.I.N.I plus interview were administered during a face-to-face or telephone interview, to assess co-morbid diagnoses. "
"In order to calculate quality-adjusted life-years (QALYs), the EuroQol (EQ-5D-3L) [48] questionnaire was administered online at all four time-points. QALYs gained over the 30-week study period were estimated by linearly interpolating EQ-5D utility over the time points and correcting for time. Utility scores were based on the Dutch tariff [49]."
" Self-reported resource use in the four weeks prior to each assessment was measured with a web-based version of the Trimbos and iMTA questionnaire on Costs associated with Psychiatric illness (TiC-P) questionnaire [50]. [...] For the main economic evaluation, costs were estimated from a societal perspective, which included all costs measured by the TiC-P. The healthcare system perspective only included direct medical costs. [...] Costs were computed by multiplying the number of units (contacts, visits, sessions) by the standard unit cost-prices (for the year 2014) as reported in the most recent Dutch guideline for economic evaluations [51]. When costs were not included in the latest version of the manual, the cost-price was derived from the previous guideline [52] and indexed to the year 2014. Medication prices were retrieved from the National Healthcare Institute (Zorginstituut Nederland) (Z-index, 2016). For each prescription, the standard dispensing fee for pharmacists of €6 was added. For bCBT patients, costs associated with the actual number of online feedback messages received were added, based on an estimated 30 minutes of therapist time per feedback message. Cumulative costs over the 30-week study period were estimated using linear interpolation."

6a-i) Online questionnaires: describe if they were validated for online use and apply CHERRIES items to describe how the questionnaires were designed/deployed

If outcomes were obtained through online questionnaires, describe if they were validated for online use and apply CHERRIES items to describe how the questionnaires were designed/deployed [9].

	1	2	3	4	5	
subitem not at all important	○	○	○	○	○	essential

Does your paper address subitem 6a-i?

Copy and paste relevant sections from manuscript text

Your answer

6a-ii) Describe whether and how “use” (including intensity of use/dosage) was defined/measured/monitored

Describe whether and how “use” (including intensity of use/dosage) was defined/measured/monitored (logins, logfile analysis, etc.). Use/adoption metrics are important process outcomes that should be reported in any ehealth trial.

	1	2	3	4	5	
subitem not at all important	○	○	○	○	○	essential

Does your paper address subitem 6a-ii?

Copy and paste relevant sections from manuscript text

Your answer

6a-iii) Describe whether, how, and when qualitative feedback from participants was obtained

Describe whether, how, and when qualitative feedback from participants was obtained (e.g., through emails, feedback forms, interviews, focus groups).

	1	2	3	4	5	
subitem not at all important	○	○	○	○	○	essential

Does your paper address subitem 6a-iii?

Copy and paste relevant sections from manuscript text

Your answer

6b) Any changes to trial outcomes after the trial commenced, with reasons

Does your paper address CONSORT subitem 6b? *

Copy and paste relevant sections from the manuscript (include quotes in quotation marks "like this" to indicate direct quotes from your manuscript), or elaborate on this item by providing additional information not in the ms, or briefly explain why the item is not applicable/relevant for your study

No changes to trial outcomes. Treatment respons was further specified: "Within the cost-effectiveness framework, the IDS-SR30 scores were used to assess treatment response, based on the reliable change (RC) index [46]. Baseline IDS-SR30 scores were subtracted from follow-up scores and subsequently divided by Sdiff (in current sample 4.78, Cronbach's alpha .84). Treatment response (reliable change) was coded as absent (RC □ -1.96) versus present (RC<-1.96). Remission was defined as the combination of an RC<-1.96 and an IDS-SR30 score below 13, indicating no depression severity on the IDS-SR30 severity index [42,47]. "

7a) How sample size was determined

NPT: When applicable, details of whether and how the clustering by care provides or centers was addressed

7a-i) Describe whether and how expected attrition was taken into account when calculating the sample size

Describe whether and how expected attrition was taken into account when calculating the sample size.

	1	2	3	4	5	
subitem not at all important	○	○	○	○	○	essential

Does your paper address subitem 7a-i?

Copy and paste relevant sections from manuscript title (include quotes in quotation marks "like this" to indicate direct quotes from your manuscript), or elaborate on this item by providing additional information not in the ms, or briefly explain why the item is not applicable/relevant for your study

"Sample size estimation was based on the probability of bCBT being cost-effective in comparison to standard CBT for various willingness-to-pay ceilings (WTP), i.e. the maximum additional financial contribution society is willing to invest in order to gain one more unit of treatment effect [57]. Through a simulation study, we determined the impact of different sample sizes on stability of the cost-effectiveness acceptability curve (CEAC) [57]. Using realistic fixed estimates of the mean and standard deviation of effects and the costs on the population level (based on Hakkaart-van Roijen et al. [58]), we simulated a large number of trials in which we systematically varied sample sizes between n=10 to n=500 per group. At a sample size of n=75 per group, we found probability estimates to converge to acceptable 75% values within the relevant range of willingness-to-pay ceilings. Therefore, we aimed for a sample size of n=150. "

7b) When applicable, explanation of any interim analyses and stopping guidelines

Does your paper address CONSORT subitem 7b? *

Copy and paste relevant sections from the manuscript (include quotes in quotation marks "like this" to indicate direct quotes from your manuscript), or elaborate on this item by providing additional information not in the ms, or briefly explain why the item is not applicable/relevant for your study

Not applicable

8a) Method used to generate the random allocation sequence

NPT: When applicable, how care providers were allocated to each trial group

Does your paper address CONSORT subitem 8a? *

Copy and paste relevant sections from the manuscript (include quotes in quotation marks "like this" to indicate direct quotes from your manuscript), or elaborate on this item by providing additional information not in the ms, or briefly explain why the item is not applicable/relevant for your study

"Allocation was stratified by mental healthcare centre using a computer-generated random number table. An independent researcher conducted the randomisation. "

8b) Type of randomisation; details of any restriction (such as blocking and block size)

Does your paper address CONSORT subitem 8b? *

Copy and paste relevant sections from the manuscript (include quotes in quotation marks "like this" to indicate direct quotes from your manuscript), or elaborate on this item by providing additional information not in the ms, or briefly explain why the item is not applicable/relevant for your study

"Allocation was stratified by mental healthcare centre using a computer-generated random number table. An independent researcher conducted the randomisation. "

9) Mechanism used to implement the random allocation sequence (such as sequentially numbered containers), describing any steps taken to conceal the sequence until interventions were assigned

Does your paper address CONSORT subitem 9? *

Copy and paste relevant sections from the manuscript (include quotes in quotation marks "like this" to indicate direct quotes from your manuscript), or elaborate on this item by providing additional information not in the ms, or briefly explain why the item is not applicable/relevant for your study

"Patients were randomly allocated to bCBT or standard CBT when they met inclusion criteria, provided a signed informed consent form and completed the full baseline assessment. Allocation was stratified by mental healthcare centre using a computer-generated random number table. An independent researcher conducted the randomisation. Patients and therapists were aware of group allocation, but allocation was concealed from outcome assessors."

10) Who generated the random allocation sequence, who enrolled participants, and who assigned participants to interventions

Does your paper address CONSORT subitem 10? *

Copy and paste relevant sections from the manuscript (include quotes in quotation marks "like this" to indicate direct quotes from your manuscript), or elaborate on this item by providing additional information not in the ms, or briefly explain why the item is not applicable/relevant for your study

"Allocation was stratified by mental healthcare centre using a computer-generated random number table. An independent researcher conducted the randomisation."

11a) If done, who was blinded after assignment to interventions (for example, participants, care providers, those assessing outcomes) and how

NPT: Whether or not administering co-interventions were blinded to group assignment

11a-i) Specify who was blinded, and who wasn’t

Specify who was blinded, and who wasn’t. Usually, in web-based trials it is not possible to blind the participants [1, 3] (this should be clearly acknowledged), but it may be possible to blind outcome assessors, those doing data analysis or those administering co-interventions (if any).

	1	2	3	4	5	
subitem not at all important	○	○	○	○	○	essential

Does your paper address subitem 11a-i? *

Copy and paste relevant sections from the manuscript (include quotes in quotation marks "like this" to indicate direct quotes from your manuscript), or elaborate on this item by providing additional information not in the ms, or briefly explain why the item is not applicable/relevant for your study

"Patients and therapists were aware of group allocation, but allocation was concealed from outcome assessors."

11a-ii) Discuss e.g., whether participants knew which intervention was the “intervention of interest” and which one was the “comparator”

Informed consent procedures (4a-ii) can create biases and certain expectations - discuss e.g., whether participants knew which intervention was the “intervention of interest” and which one was the “comparator”.

	1	2	3	4	5	
subitem not at all important	○	○	○	○	○	essential

Does your paper address subitem 11a-ii?

Copy and paste relevant sections from the manuscript (include quotes in quotation marks "like this" to indicate direct quotes from your manuscript), or elaborate on this item by providing additional information not in the ms, or briefly explain why the item is not applicable/relevant for your study

Your answer

11b) If relevant, description of the similarity of interventions

(this item is usually not relevant for ehealth trials as it refers to similarity of a placebo or sham intervention to a active medication/intervention)

Does your paper address CONSORT subitem 11b? *

Copy and paste relevant sections from the manuscript (include quotes in quotation marks "like this" to indicate direct quotes from your manuscript), or elaborate on this item by providing additional information not in the ms, or briefly explain why the item is not applicable/relevant for your study

Similarities in both interventions are discussed. "Both interventions consisted of cognitive behavioural depression treatment. The content of CBT was based on the standard treatment manual [39–41], which recommends 15 to 20 weekly sessions focusing on psycho-education, behavioural activation, cognitive restructuring and relapse prevention. Patients who completed 14 sessions or more (75% of 18 sessions) were considered treatment completers. Parallel pharmacological treatment was allowed. "

12a) Statistical methods used to compare groups for primary and secondary outcomes

NPT: When applicable, details of whether and how the clustering by care providers or centers was addressed

Does your paper address CONSORT subitem 12a? *

Copy and paste relevant sections from the manuscript (include quotes in quotation marks "like this" to indicate direct quotes from your manuscript), or elaborate on this item by providing additional information not in the ms, or briefly explain why the item is not applicable/relevant for your study

"For descriptive purposes, clinical and cost outcomes were first examined in separate Linear Mixed-effect Models. In order to account for missing data and the correlation between follow-up time-points, Linear Mixed-effect Models (LMM) with restricted maximum likelihood (REML) were used to estimate the treatment effects, EQ-5D utility gained and cumulative costs across time. The models included both fixed and random effects, which allowed for estimation on a patient-level of how far each patient diverged from the fixed (group-level) effect over time [53,54]. Main outcome measures (i.e. IDS-SR depression severity, reliable change, MINI depression status, EQ-5D QALYs and costs) were evaluated in separate mixed models. Time was included as a categorical variable (0, 10, 20 and 30 weeks). For diagnosis of depressive episodes (present versus absent) and reliable improvement of depression severity (improvement versus no improvement) a Logistic Mixed-effects Model was fitted. In these models, the random intercept was dropped from the model, as all patients started at the same baseline value (a current diagnosis of depression).

For the cost-effectiveness analyses, baseline adjusted Linear Mixed-effect Models were estimated with group as independent variable, and societal costs, direct medical costs, and reliable change in depression severity as dependent variables and a random effect across individuals. For dichotomous outcomes, Linear Mixed-effect Models were fitted along with the logistic models in order to estimate risk difference (RD) and calculate the ICERs. EQ-5D QALYs gained during the study were used as dependent variable for the cost-utility analysis. Societal costs during the study period were used as the main cost outcome. Direct healthcare costs were examined as a separate cost outcome in order to conduct the health-economic evaluation from the healthcare system perspective. Ninety-five percent uncertainty intervals around LMM regression estimates for group were estimated with 5,000 probabilistic Monte Carlo simulations, running all mixed models simultaneously.

The resulting pairs of (bootstrapped) cost and effect or utility estimates were plotted on cost-effectiveness and cost-utility planes. The y-axis represents relative costs and the x-axis relative effects associated with bCBT versus standard CBT. The axes divide the plane in four quadrants: North-East (NE) quadrant: bCBT is more expensive and more effective than standard CBT; North-West (NW) quadrant: the new intervention (bCBT) is more expensive and less effective than status quo (standard CBT); the South-West (SW) quadrant: bCBT is less expensive and less effective than standard CBT and South-East (SE) quadrant: bCBT is less expensive and more effective than standard CBT. Cost-effectiveness acceptability curves (CEACs) were estimated to assess the probability of bCBT being cost-effective given various willingness-to-pay (WTP)

ceilings.
The mixed models were estimated using the lmer and glmer functions from the lme4 package (version 1.1-15) [55] using R software (version 3.4.4) [56]. Combined cost-effectiveness and cost-utility analyses were performed in Stata (version 14.2, StataCorp LP)."

12a-i) Imputation techniques to deal with attrition / missing values

Imputation techniques to deal with attrition / missing values: Not all participants will use the intervention/comparator as intended and attrition is typically high in ehealth trials. Specify how participants who did not use the application or dropped out from the trial were treated in the statistical analysis (a complete case analysis is strongly discouraged, and simple imputation techniques such as LOCF may also be problematic [4]).

	1	2	3	4	5	
subitem not at all important	○	○	○	○	○	essential

Does your paper address subitem 12a-i? *

Copy and paste relevant sections from the manuscript (include quotes in quotation marks "like this" to indicate direct quotes from your manuscript), or elaborate on this item by providing additional information not in the ms, or briefly explain why the item is not applicable/relevant for your study

"In order to account for missing data and the correlation between follow-up time-points, Linear Mixed-effect Models (LMM) with restricted maximum likelihood (REML) were used to estimate the treatment effects, EQ-5D utility gained and cumulative costs across time"

12b) Methods for additional analyses, such as subgroup analyses and adjusted analyses

Does your paper address CONSORT subitem 12b? *

Copy and paste relevant sections from the manuscript (include quotes in quotation marks "like this" to indicate direct quotes from your manuscript), or elaborate on this item by providing additional information not in the ms, or briefly explain why the item is not applicable/relevant for your study

"Two post-hoc sensitivity analyses were conducted. First, a Linear Mixed-effect Model was estimated for societal costs excluding the costs of in-patient care (both in general hospitals and mental healthcare), because while these events are rare, they can act as influential outliers due to the high costs associated with in-patient care. Second, direct non-medical costs associated with absenteeism and presenteeism were evaluated as a separate cost outcome to assess their contribution to the overall societal costs as evaluated in the main analysis. "

X26) REB/IRB Approval and Ethical Considerations [recommended as subheading under "Methods"] (not a CONSORT item)

X26-i) Comment on ethics committee approval

	1	2	3	4	5	
subitem not at all important	○	○	○	○	○	essential

Does your paper address subitem X26-i?

Copy and paste relevant sections from the manuscript (include quotes in quotation marks "like this" to indicate direct quotes from your manuscript), or elaborate on this item by providing additional information not in the ms, or briefly explain why the item is not applicable/relevant for your study

"The trial was registered in the Netherlands Trial Register (registration number NTR4650) and was approved by the Medical Ethics Committee of the VU University Medical Centre (registration number 2014.191)."

x26-ii) Outline informed consent procedures

Outline informed consent procedures e.g., if consent was obtained offline or online (how? Checkbox, etc.?), and what information was provided (see 4a-ii). See [6] for some items to be included in informed consent documents.

	1	2	3	4	5	
subitem not at all important	○	○	○	○	○	essential

Does your paper address subitem X26-ii?

Copy and paste relevant sections from the manuscript (include quotes in quotation marks "like this" to indicate direct quotes from your manuscript), or elaborate on this item by providing additional information not in the ms, or briefly explain why the item is not applicable/relevant for your study

Consent was obtained offline: "Patients were randomly allocated to bCBT or standard CBT when they met inclusion criteria, provided a signed informed consent form and completed the full baseline assessment."

X26-iii) Safety and security procedures

Safety and security procedures, incl. privacy considerations, and any steps taken to reduce the likelihood or detection of harm (e.g., education and training, availability of a hotline)

	1	2	3	4	5	
subitem not at all important	○	○	○	○	○	essential

Does your paper address subitem X26-iii?

Copy and paste relevant sections from the manuscript (include quotes in quotation marks "like this" to indicate direct quotes from your manuscript), or elaborate on this item by providing additional information not in the ms, or briefly explain why the item is not applicable/relevant for your study

Your answer

RESULTS

13a) For each group, the numbers of participants who were randomly assigned, received intended treatment, and were analysed for the primary outcome

NPT: The number of care providers or centers performing the intervention in each group and the number of patients treated by each care provider in each center

Does your paper address CONSORT subitem 13a? *

Copy and paste relevant sections from the manuscript (include quotes in quotation marks "like this" to indicate direct quotes from your manuscript), or elaborate on this item by providing additional information not in the ms, or briefly explain why the item is not applicable/relevant for your study

A figure is added to the MS showing the patient flow through both treatment arms.

13b) For each group, losses and exclusions after randomisation, together with reasons

Does your paper address CONSORT subitem 13b? (NOTE: Preferably, this is shown in a CONSORT flow diagram) *

Copy and paste relevant sections from the manuscript (include quotes in quotation marks "like this" to indicate direct quotes from your manuscript), or elaborate on this item by providing additional information not in the ms, or briefly explain why the item is not applicable/relevant for your study

Information is provided in Figure 1 in the MS
"Between August 2014 and May 2016, 103 patients were randomised to bCBT (n=54) or standard CBT (n=49). One patient withdrew from the study participation before commencing treatment due to starting another treatment elsewhere. "
"Overall, the M.I.N.I plus diagnostic interview could be administered to 85 patients (83%) at 10 weeks, 84 patients (82%) at 20 weeks, and 76 patients (75%) at 30 weeks. The online self-report questionnaires were completed by 77 patients at 10 weeks (76%), 65 patients (64%) at 20 weeks, and 65 patients (64%) at 30 weeks."

13b-i) Attrition diagram

Strongly recommended: An attrition diagram (e.g., proportion of participants still logging in or using the intervention/comparator in each group plotted over time, similar to a survival curve) or other figures or tables demonstrating usage/dose/engagement.

	1	2	3	4	5	
subitem not at all important	○	○	○	○	○	essential

Does your paper address subitem 13b-i?

Copy and paste relevant sections from the manuscript or cite the figure number if applicable (include quotes in quotation marks "like this" to indicate direct quotes from your manuscript), or elaborate on this item by providing additional information not in the ms, or briefly explain why the item is not applicable/relevant for your study

Figure 1 provides information on the number of sessions completed in both groups. Additional information is provided in the text
"Ninety-seven patients (95%) started treatment, receiving at least one session. In bCBT, the average number of sessions matched the planned number, with a total of 19 sessions (SD=8.1, range 0 to 31). On average, bCBT consisted of 9 online sessions (SD=4.0, range 0 to 16) and 10 face-to-face sessions (SD=4.6, range 0 to 16). Per-patient online feedback was provided 8 (SD=3.8, range 0 to 15) times on average. Based on the cut-off of 14 sessions (75% of the optimal bCBT protocol of 19 sessions), 43 bCBT patients (81%) were considered treatment completers. When the 75% completion criterion is applied to online and face-to-face sessions separately (at least 7 of each), 40 bCBT patients (75%) were considered completers. Seven patients (13%) completed the full bCBT protocol within 10 weeks.
In standard CBT, the mean number of sessions was 13 (SD=6.3, range 0 to 27), which was less than planned (15 to 18) within the time-frame of the current study. Based on the cut-off of 14 sessions, 22 patients (45%) were considered treatment completers. Four patients (8%) completed the standard CBT protocol (16 to 20 sessions) within 20 weeks.
Compared to patients in the standard CBT group, bCBT patients received significantly more sessions in total (19 versus 13, t(100)=-4.09, p<.001), but significantly fewer face-to-face sessions at the clinic (10 versus 13, t(100)=3.07, p=.003). As expected based on the treatment protocol, mean treatment duration was significantly shorter in bCBT than in standard CBT, with an average of 19 weeks (SD=12.6) versus 33 weeks (SD=23.0), t(100)=3.91, p<.001. When therapist time spent on online feedback was included, the combined amount of direct and indirect therapist time did not differ between groups with an average of 14.0 hours for bCBT (SD=6.2) and 13.3 hours for standard CBT (SD=6.3), t(100)=-.55, p=.582)."

14a) Dates defining the periods of recruitment and follow-up

Does your paper address CONSORT subitem 14a? *

Copy and paste relevant sections from the manuscript (include quotes in quotation marks "like this" to indicate direct quotes from your manuscript), or elaborate on this item by providing additional information not in the ms, or briefly explain why the item is not applicable/relevant for your study

"Between August 2014 and May 2016, 103 patients were randomised to bCBT (n=54) or standard CBT (n=49)"

14a-i) Indicate if critical “secular events” fell into the study period

Indicate if critical “secular events” fell into the study period, e.g., significant changes in Internet resources available or “changes in computer hardware or Internet delivery resources”

	1	2	3	4	5	
subitem not at all important	○	○	○	○	○	essential

Does your paper address subitem 14a-i?

Copy and paste relevant sections from the manuscript (include quotes in quotation marks "like this" to indicate direct quotes from your manuscript), or elaborate on this item by providing additional information not in the ms, or briefly explain why the item is not applicable/relevant for your study

Your answer

14b) Why the trial ended or was stopped (early)

Does your paper address CONSORT subitem 14b? *

Copy and paste relevant sections from the manuscript (include quotes in quotation marks "like this" to indicate direct quotes from your manuscript), or elaborate on this item by providing additional information not in the ms, or briefly explain why the item is not applicable/relevant for your study

"The full intention-to-treat (ITT) sample consists of 102 patients (bCBT=53, standard CBT=49). This sample size was smaller than the initial goal of 150 patients [38]. The recruitment and screening of sufficient patients to randomise 150 patients, proved to be unfeasible within the fixed time-frame of the current study. Fewer patients were referred to specialised services than expected. This was partly due to a nationwide reorganisation of Dutch mental healthcare, which meant that a larger proportion of depressed patients were treated in primary mental healthcare, rather than specialised care. "

15) A table showing baseline demographic and clinical characteristics for each group

NPT: When applicable, a description of care providers (case volume, qualification, expertise, etc.) and centers (volume) in each group

Does your paper address CONSORT subitem 15? *

Copy and paste relevant sections from the manuscript (include quotes in quotation marks "like this" to indicate direct quotes from your manuscript), or elaborate on this item by providing additional information not in the ms, or briefly explain why the item is not applicable/relevant for your study

Such a table is included in the MS.

15-i) Report demographics associated with digital divide issues

In ehealth trials it is particularly important to report demographics associated with digital divide issues, such as age, education, gender, social-economic status, computer/Internet/ehealth literacy of the participants, if known.

	1	2	3	4	5	
subitem not at all important	○	○	○	○	○	essential

Does your paper address subitem 15-i? *

Copy and paste relevant sections from the manuscript (include quotes in quotation marks "like this" to indicate direct quotes from your manuscript), or elaborate on this item by providing additional information not in the ms, or briefly explain why the item is not applicable/relevant for your study

Information is provided on age, education, gender, and social-economic variables

16) For each group, number of participants (denominator) included in each analysis and whether the analysis was by original assigned groups

16-i) Report multiple “denominators” and provide definitions

Report multiple “denominators” and provide definitions: Report N’s (and effect sizes) “across a range of study participation [and use] thresholds” [1], e.g., N exposed, N consented, N used more than x times, N used more than y weeks, N participants “used” the intervention/comparator at specific pre-defined time points of interest (in absolute and relative numbers per group). Always clearly define “use” of the intervention.

	1	2	3	4	5	
subitem not at all important	○	○	○	○	○	essential

Does your paper address subitem 16-i? *

Copy and paste relevant sections from the manuscript (include quotes in quotation marks "like this" to indicate direct quotes from your manuscript), or elaborate on this item by providing additional information not in the ms, or briefly explain why the item is not applicable/relevant for your study

Information on the uptake is provided in figure 1 and in the MS (see, 13b-i)

16-ii) Primary analysis should be intent-to-treat

Primary analysis should be intent-to-treat, secondary analyses could include comparing only "users", with the appropriate caveats that this is no longer a randomized sample (see 18-i).

	1	2	3	4	5	
subitem not at all important	○	○	○	○	○	essential

Does your paper address subitem 16-ii?

Copy and paste relevant sections from the manuscript (include quotes in quotation marks "like this" to indicate direct quotes from your manuscript), or elaborate on this item by providing additional information not in the ms, or briefly explain why the item is not applicable/relevant for your study

Your answer

17a) For each primary and secondary outcome, results for each group, and the estimated effect size and its precision (such as 95% confidence interval)

Does your paper address CONSORT subitem 17a? *

Copy and paste relevant sections from the manuscript (include quotes in quotation marks "like this" to indicate direct quotes from your manuscript), or elaborate on this item by providing additional information not in the ms, or briefly explain why the item is not applicable/relevant for your study

Information on outcomes is provided in text and in Tables.

17a-i) Presentation of process outcomes such as metrics of use and intensity of use

In addition to primary/secondary (clinical) outcomes, the presentation of process outcomes such as metrics of use and intensity of use (dose, exposure) and their operational definitions is critical. This does not only refer to metrics of attrition (13-b) (often a binary variable), but also to more continuous exposure metrics such as “average session length”. These must be accompanied by a technical description how a metric like a “session” is defined (e.g., timeout after idle time) [1] (report under item 6a).

	1	2	3	4	5	
subitem not at all important	○	○	○	○	○	essential

Does your paper address subitem 17a-i?

Copy and paste relevant sections from the manuscript (include quotes in quotation marks "like this" to indicate direct quotes from your manuscript), or elaborate on this item by providing additional information not in the ms, or briefly explain why the item is not applicable/relevant for your study

Your answer

17b) For binary outcomes, presentation of both absolute and relative effect sizes is recommended

Does your paper address CONSORT subitem 17b? *

Copy and paste relevant sections from the manuscript (include quotes in quotation marks "like this" to indicate direct quotes from your manuscript), or elaborate on this item by providing additional information not in the ms, or briefly explain why the item is not applicable/relevant for your study

Yes, information is included in text and in Tables.

18) Results of any other analyses performed, including subgroup analyses and adjusted analyses, distinguishing pre-specified from exploratory

Does your paper address CONSORT subitem 18? *

Copy and paste relevant sections from the manuscript (include quotes in quotation marks "like this" to indicate direct quotes from your manuscript), or elaborate on this item by providing additional information not in the ms, or briefly explain why the item is not applicable/relevant for your study

Yes.

18-i) Subgroup analysis of comparing only users

A subgroup analysis of comparing only users is not uncommon in ehealth trials, but if done, it must be stressed that this is a self-selected sample and no longer an unbiased sample from a randomized trial (see 16-iii).

	1	2	3	4	5	
subitem not at all important	○	○	○	○	○	essential

Does your paper address subitem 18-i?

Copy and paste relevant sections from the manuscript (include quotes in quotation marks "like this" to indicate direct quotes from your manuscript), or elaborate on this item by providing additional information not in the ms, or briefly explain why the item is not applicable/relevant for your study

Your answer

19) All important harms or unintended effects in each group

(for specific guidance see CONSORT for harms)

Does your paper address CONSORT subitem 19? *

Copy and paste relevant sections from the manuscript (include quotes in quotation marks "like this" to indicate direct quotes from your manuscript), or elaborate on this item by providing additional information not in the ms, or briefly explain why the item is not applicable/relevant for your study

"There were no patients who consecutively reported deterioration at all follow-up assessments. No deaths occurred during the study. One depression-related, but not treatment-related, serious adverse event occurred in the standard CBT group, where one person self-harmed. "

19-i) Include privacy breaches, technical problems

Include privacy breaches, technical problems. This does not only include physical “harm” to participants, but also incidents such as perceived or real privacy breaches [1], technical problems, and other unexpected/unintended incidents. “Unintended effects” also includes unintended positive effects [2].

	1	2	3	4	5	
subitem not at all important	○	○	○	○	○	essential

Does your paper address subitem 19-i?

Copy and paste relevant sections from the manuscript (include quotes in quotation marks "like this" to indicate direct quotes from your manuscript), or elaborate on this item by providing additional information not in the ms, or briefly explain why the item is not applicable/relevant for your study

Your answer

19-ii) Include qualitative feedback from participants or observations from staff/researchers

Include qualitative feedback from participants or observations from staff/researchers, if available, on strengths and shortcomings of the application, especially if they point to unintended/unexpected effects or uses. This includes (if available) reasons for why people did or did not use the application as intended by the developers.

	1	2	3	4	5	
subitem not at all important	○	○	○	○	○	essential

Does your paper address subitem 19-ii?

Copy and paste relevant sections from the manuscript (include quotes in quotation marks "like this" to indicate direct quotes from your manuscript), or elaborate on this item by providing additional information not in the ms, or briefly explain why the item is not applicable/relevant for your study

Your answer

DISCUSSION

22) Interpretation consistent with results, balancing benefits and harms, and considering other relevant evidence

NPT: In addition, take into account the choice of the comparator, lack of or partial blinding, and unequal expertise of care providers or centers in each group

22-i) Restate study questions and summarize the answers suggested by the data, starting with primary outcomes and process outcomes (use)

Restate study questions and summarize the answers suggested by the data, starting with primary outcomes and process outcomes (use).

	1	2	3	4	5	
subitem not at all important	○	○	○	○	○	essential

Does your paper address subitem 22-i? *

Copy and paste relevant sections from the manuscript (include quotes in quotation marks "like this" to indicate direct quotes from your manuscript), or elaborate on this item by providing additional information not in the ms, or briefly explain why the item is not applicable/relevant for your study

"This pilot randomised controlled trial focused on depression treatment in Dutch routine specialised mental healthcare and compared the costs and effects of blended CBT (bCBT) for major depressive disorder to standard (evidence-based) face-to-face CBT during a 30-week time-frame. The study examined whether bCBT can lead to comparable clinical effects as standard CBT, at lower costs. To the best of our knowledge, our study was the first to explore the cost-effectiveness of integrated (rather than add-on) blended depression treatment in routine mental health care and compare this blended therapy to existing, evidence-based, face-to-face CBT.

Principal findings
The results suggest that bCBT for depression can lead to comparable costs and clinical effects as standard CBT. In both treatment groups severity of depressive symptoms decreased, the probability of having a depression diagnosis lessened, and quality of life improved. When costs and effects were combined, results were mixed. From a societal perspective, which includes productivity losses, bCBT had a low probability of being cost-effective compared to standard CBT. From a healthcare provider perspective bCBT had a high probability of being cost-effective for treatment response (reliable change in depression severity) and quality-adjusted life-years (QALYs, cost-utility), but not for depression diagnosis (depressive episodes).
Results show that bCBT patients received significantly fewer face-to-face sessions than standard CBT patients (10 versus 13 sessions). In line with our expectations, bCBT also led to a shorter treatment duration (19 versus 33 weeks). After 10 weeks of treatment, 51% of bCBT patients completed 75% or more of the treatment protocol (at least 14 sessions in total), versus 5% in the CBT group. After 30 weeks, the percentage of treatment completers rose to 85% for bCBT and 45% for standard CBT. Combined with therapist-time required for online feedback, bCBT and standard CBT unexpectedly required similar per-patient time investments from therapists (14 versus 13 hours). This was mainly driven by the mean number of sessions in standard CBT, which was considerably lower than expected based on the protocol (13 versus 18 sessions). "

22-ii) Highlight unanswered new questions, suggest future research

Highlight unanswered new questions, suggest future research.

	1	2	3	4	5	
subitem not at all important	◯	◯	◯	◯	◯	essential

Does your paper address subitem 22-ii?

Copy and paste relevant sections from the manuscript (include quotes in quotation marks "like this" to indicate direct quotes from your manuscript), or elaborate on this item by providing additional information not in the ms, or briefly explain why the item is not applicable/relevant for your study

"Future studies should focus on hypothesis testing using a non-inferiority design" "Further research seems warranted, focussing on optimising the clinical effects of blended treatment, as well as cost-impact"

20) Trial limitations, addressing sources of potential bias, imprecision, and, if relevant, multiplicity of analyses

20-i) Typical limitations in ehealth trials

Typical limitations in ehealth trials: Participants in ehealth trials are rarely blinded. Ehealth trials often look at a multiplicity of outcomes, increasing risk for a Type I error. Discuss biases due to non-use of the intervention/usability issues, biases through informed consent procedures, unexpected events.

	1	2	3	4	5	
subitem not at all important	◯	◯	◯	◯	◯	essential

Does your paper address subitem 20-i? *

Copy and paste relevant sections from the manuscript (include quotes in quotation marks "like this" to indicate direct quotes from your manuscript), or elaborate on this item by providing additional information not in the ms, or briefly explain why the item is not applicable/relevant for your study

"There are a number of factors that need to be considered when interpreting the findings. First, the sample size was lower than anticipated. This was mainly due to time constraints of conducting the study. The achieved sample is considered adequate for the central aim of the study, which was to guide further development and research in blended CBT. Future studies should focus on hypothesis testing using a non-inferiority design.
Second, the current sample was characterised by a high level of heterogeneity, both in clinical and cost characteristics. On the one hand this is a positive aspect, as it is representative of the population in routine specialised mental healthcare. On the other hand, it led to some baseline imbalances between groups. This is most apparent in the average direct medical and non-medical costs patients reported in the month before baseline, which were higher in the bCBT group than the standard CBT group. This was primarily caused by the fact that in the blended group three patients reported having been admitted to an in-patient psychiatric ward prior to baseline assessment, versus no patients in the standard CBT group. The imbalances at baseline increased the uncertainty around the cost and effectiveness estimates, especially at 30-week follow-up. This is reflected in the analyses concerning treatment response, quality of life and direct medical costs (healthcare provider perspective). The models that included time were in favour of the standard CBT group, while the models that did not include time were in favour of blended CBT. It is important to keep in mind that all findings were non-significant and varied around zero, signifying no differences between groups. "

21) Generalisability (external validity, applicability) of the trial findings

NPT: External validity of the trial findings according to the intervention, comparators, patients, and care providers or centers involved in the trial

21-i) Generalizability to other populations

Generalizability to other populations: In particular, discuss generalizability to a general Internet population, outside of a RCT setting, and general patient population, including applicability of the study results for other organizations

	1	2	3	4	5	
subitem not at all important	○	○	○	○	○	essential

Does your paper address subitem 21-i?

Copy and paste relevant sections from the manuscript (include quotes in quotation marks "like this" to indicate direct quotes from your manuscript), or elaborate on this item by providing additional information not in the ms, or briefly explain why the item is not applicable/relevant for your study

The trial focussed on patients in routine practice, aiming to increase generalisability

21-ii) Discuss if there were elements in the RCT that would be different in a routine application setting

Discuss if there were elements in the RCT that would be different in a routine application setting (e.g., prompts/reminders, more human involvement, training sessions or other co-interventions) and what impact the omission of these elements could have on use, adoption, or outcomes if the intervention is applied outside of a RCT setting.

	1	2	3	4	5	
subitem not at all important	○	○	○	○	○	essential

Does your paper address subitem 21-ii?

Copy and paste relevant sections from the manuscript (include quotes in quotation marks "like this" to indicate direct quotes from your manuscript), or elaborate on this item by providing additional information not in the ms, or briefly explain why the item is not applicable/relevant for your study

Your answer

OTHER INFORMATION

23) Registration number and name of trial registry

Does your paper address CONSORT subitem 23? *

Copy and paste relevant sections from the manuscript (include quotes in quotation marks "like this" to indicate direct quotes from your manuscript), or elaborate on this item by providing additional information not in the ms, or briefly explain why the item is not applicable/relevant for your study

The trial was registered in the Netherlands Trial Register (registration number NTR4650)

24) Where the full trial protocol can be accessed, if available

Does your paper address CONSORT subitem 24? *

Cite a Multimedia Appendix, other reference, or copy and paste relevant sections from the manuscript (include quotes in quotation marks "like this" to indicate direct quotes from your manuscript), or elaborate on this item by providing additional information not in the ms, or briefly explain why the item is not applicable/relevant for your study

The trial protocol has been published
Kooistra LC, Wiersma JE, Ruwaard J, van Oppen P, Smit F, Lokkerbol J, et al. Blended vs. face-to-face cognitive behavioural treatment for major depression in specialized mental health care: study protocol of a randomized controlled cost-effectiveness trial. BMC Psychiatry. 2014;14(1):1–11.

25) Sources of funding and other support (such as supply of drugs), role of funders

Does your paper address CONSORT subitem 25? *

Copy and paste relevant sections from the manuscript (include quotes in quotation marks "like this" to indicate direct quotes from your manuscript), or elaborate on this item by providing additional information not in the ms, or briefly explain why the item is not applicable/relevant for your study

"This study was funded by ZonMw (project number 837001007)."

X27) Conflicts of Interest (not a CONSORT item)

X27-i) State the relation of the study team towards the system being evaluated

In addition to the usual declaration of interests (financial or otherwise), also state the relation of the study team towards the system being evaluated, i.e., state if the authors/evaluators are distinct from or identical with the developers/sponsors of the intervention.

	1	2	3	4	5	
subitem not at all important	○	○	○	○	○	essential

Does your paper address subitem X27-i?

Copy and paste relevant sections from the manuscript (include quotes in quotation marks "like this" to indicate direct quotes from your manuscript), or elaborate on this item by providing additional information not in the ms, or briefly explain why the item is not applicable/relevant for your study

"All authors declare no competing interests."

About the CONSORT EHEALTH checklist

As a result of using this checklist, did you make changes in your manuscript? *

- ( ) yes, major changes
- (•) yes, minor changes
- ( ) no

What were the most important changes you made as a result of using this checklist?

Your answer

How much time did you spend on going through the checklist INCLUDING making changes in your manuscript *

3 hours

As a result of using this checklist, do you think your manuscript has improved? *

- ( ) yes
- (•) no
- ( ) Other:

Would you like to become involved in the CONSORT EHEALTH group?

This would involve for example becoming involved in participating in a workshop and writing an "Explanation and Elaboration" document

- yes
- no
- Other:

Any other comments or questions on CONSORT EHEALTH

Your answer

STOP - Save this form as PDF before you click submit

To generate a record that you filled in this form, we recommend to generate a PDF of this page (on a Mac, simply select "print" and then select "print as PDF") before you submit it.

When you submit your (revised) paper to JMIR, please upload the PDF as supplementary file.

Don't worry if some text in the textboxes is cut off, as we still have the complete information in our database. Thank you!

Final step: Click submit !

Click submit so we have your answers in our database!

SUBMIT

Never submit passwords through Google Forms.

This content is neither created nor endorsed by Google. Report Abuse - Terms of Service

Google Forms
